# Identification and genetic characterization of mitochondrial citrate transporters in *Aspergillus niger*

**DOI:** 10.3389/fmicb.2022.1009491

**Published:** 2022-09-13

**Authors:** Wei Cao, Licheng Zhang, Liu Wu, Mingyi Zhang, Jiao Liu, Zhoujie Xie, Hao Liu

**Affiliations:** ^1^MOE Key Laboratory of Industrial Fermentation Microbiology, College of Biotechnology, Tianjin University of Science and Technology, Tianjin, China; ^2^Tianjin Engineering Research Center of Microbial Metabolism and Fermentation Process Control, Tianjin University of Science and Technology, Tianjin, China; ^3^National Technology Innovation Center of Synthetic Biology, Tianjin, China

**Keywords:** citric acid, mitochondrial citrate transporter, transport, genetic characterization, *Aspergillus niger*

## Abstract

*Aspergillus niger* is a major cell factory for citric acid production, and the process of citrate export from mitochondria to cytoplasm is predicted to be one of rate-limiting steps in citric acid accumulation. Currently, the mitochondrial citrate transporters (Ctps) in *A. niger* are not fully characterized. Here, six putative Ctp encoding genes (*ctpA* to *ctpF*) were identified based on their homology with a mitochondrial citrate transporter *Sc*Ctp1 from *Saccharomyces cerevisiae*. Disruption of individual *ctpA* to *ctpF* caused varying degrees of decline in citric acid accumulation at different fermentation stages, whereas a mutant strain S1696 with disruption of all six *ctp*s showed complete loss of citiric acid production. S1696 also exhibited delayed growth, reduced conidia formation, and decreased pigmentogenesis. Exogenous addition of citrate partially restored the conidia formation and pigmentogenesis in S1696 mutant. Reintroduction of individual *ctp*s (*ctpA* to *ctpF*) into S1696 at the *amyA* locus showed that *ctpA*, *ctpB*, and *ctpD* restored the citric acid titers to 88.5, 93.8, and 94.6% of the parent strain, respectively. Additionally, the formation of conidia and pigment production was partially restored after reintroduction of *ctpA*, *ctpB*, or *ctpD*. Overexpression of respective *ctpA*, *ctpB*, and *ctpD* in the parent strain resulted in increases in citric acid accumulation by 32.8, 19.3, and 24.2%, respectively. These results demonstrate that CtpA, CtpB, and CtpD play important roles in citric acid transport across the mitochondrial membrane and function in a redundant manner. Enhancement of citric acid transport process can serve as a target for boosting citric acid accumulation in *A. niger*.

## Introduction

Citric acid is an intermediate metabolite of the tricarboxylic acid (TCA) cycle and has important commercial value. Due to its safety, good flavor, high solubility, metal-chelating, and buffering ability, citric acid is extensively used in diverse fields such as food, medicine, detergent, cosmetics (Soccol et al., 2006). The worldwide market of citric acid is estimated to reach USD3.6 billion by 2025 (Mores et al., 2021).

Although several bacteria and yeasts have been found to accumulate citric acid, the performance of most strains cannot meet the needs of commercial production. Currently, *Aspergillus niger-*based fermentation is the major process for industrial production of citric acid (Tong et al., 2019; Behera, 2020) because production strains of the fungus showed excellent yields with few by-products and the good biosafety, abundant extracellular hydrolase systems for the use of cheap industrial and agricultural raw materials. High levels of citric acid accumulation by *A. niger* depend on optimization of fermentation conditions including: (1) the carbon source must be quickly available and the concentration must be higher than 5%; (2) the nitrogen sources in the medium should be able to cause a pH decrease after utilization; (3) the phosphate concentrations should be suboptimal; and (4) trace-metal-ion (especially Mn^2+^) addition should be limited and pH needs to be maintained at < 3 (Karaffa and Kubicek, 2003; Behera, 2020).

Many studies have attempted to explain the molecular mechanism of high citric acid production by *A. niger* from different perspectives, especially the activity and regulation of various enzymes related to citric acid synthesis in the primary metabolism (Behera, 2020). Given that citric acid accumulation results from an interplay of multiple factors, further mining and analysis of the factors that promote citric acid accumulation could serve as a powerful tool for the metabolic engineering of more efficient production strains of *A. niger.* Through a quantitative model of carbohydrate degradation and oxalacetate formation, three pivotal steps for citric acid accumulation have been characterized, namely, sugar uptake and phosphorylation, citrate export from mitochondria to cytoplasm, and subsequent secretion outside the cell (Torres, 1994a, 1994b). Two kinds of glucose transporters, i.e., low- and high-affinity types, have been identified and their unique physiological roles in citric acid production have been elucidated in *A. niger* (Torres, 1994b; Torres et al., 1996; Sloothaak et al., 2015; Yin et al., 2017). Recently, through homology search and transcriptomic data analysis, a major citrate transporter, CexA, responsible for exporting citric acid from cytoplasm to extracellular medium has been identified by two independent research teams (Odoni et al., 2019; Steiger et al., 2019). Its impact on glucose transport, glycolysis, and citric acid accumulation was analyzed (Nakamura et al., 2020; Xu et al., 2020).

A mitochondrial citrate transporter CTP for the transport of citric acid from mitochondria to cytoplasm was identified in rat liver cells and found to be involved in the promiscuous antiport of citrate with other tricarboxylates, dicarboxylates, and phosphoenolpyruvates (Palmieri et al., 1972; Bisaccia et al., 1989; Palmieri, 1994). By contrast, the citrate transporter *Sc*Ctp1 identified in *Saccharomyces cerevisiae* showed a much higher substrate specificity than CTPs from higher eukaryotic organisms (Kaplan et al., 1995), and no obvious phenotype was found in Δ*ScCTP1* mutants, suggesting that other unidentified mitochondrial transporter protein may be involved in this process (Kaplan et al., 1996). During the shochu brewing process, citric acid production by *Aspergillus luchuensis* mut. *Kawachii* is a typical brewing characteristic. CtpA and YhmA in *A. kawachii* has been identified as mitochondrial citrate transporters with different counter substrates that participate in acetyl-CoA generation in the cytoplasm (Kadooka et al., 2019). In *A. niger* WU-2223 l, two putative Ctp encoding genes, *ctpA* and *ctpB*, were found, but no transcripts of *ctpB* were detected under all cultivation conditions examined. *ctpA* disruption was found to cause growth delay, low spore germination rates, and low citric acid accumulation only in the early-log phase, indicating that CtpA is not the sole mitochondrial citrate transporter and that other mitochondrial transporters may participate in citric acid accumulation (Kirimura et al., 2016).

The present study aimed to comprehensively characterize the putative genes of mitochondrial citrate transporters in *A. niger* and evaluate their roles in citric acid accumulation. Using yeast *Sc*Ctp1 as template, 6 possible genes encoding mitochondrial citrate transporters were identified in the genome databases of *A. niger* through homology search and domain analyses and their roles in citric acid accumulation were validated. Moreover, strains with elevated citric acid production were constructed by overexpression of Ctps in *A. niger*.

## Materials and methods

### Strains and culture conditions

*Aspergillus niger* strain S422 (Table 1) derived from *A. niger* ATCC 1015 was used as parent strain in this study. Potato dextrose agar (PDA) was used for *A. niger* spore preparation, complete medium (CM) was used for transformant screening, and minimal medium (MM) was used for gene knock-out phenotype screening as previously described (Cao et al., 2020). *Escherichia coli* JM109 used as a host for plasmid construction, and *Agrobacterium tumefaciens* AGL-1 used for the genetic transformation of *A. niger* were cultivated in LB medium at 37 and 28°C, respectively (Xu et al., 2019). All *A. niger* strains used in this study are listed in Table 1.

**Table 1:** Strains and plasmids used in this study.

	Genotype/description	Sources
Strains		
S422	Tet-On::*cre*, Δ*oahA*	Xu et al. (2019)
S838	Tet-On::*cre*, Δ*oahA*, Δ*ctpA*	This study
S836	Tet-On::*cre*, Δ*oahA*, Δ*ctpB*	This study
S1152	Tet-On::*cre*, Δ*oahA*, Δ*ctpC*	This study
S865	Tet-On::*cre*, Δ*oahA*, Δ*ctpD*	This study
S866	Tet-On::*cre*, Δ*oahA*, Δ*ctpE*	This study
S938	Tet-On::*cre*, Δ*oahA*, Δ*ctpF*	This study
S1696	Tet-On::*cre*, Δ*oahA*, Δ*ctpA-F*	This study
S2152	Tet-On::*cre*, Δ*oahA*, Δ*ctpA*, Δ*ctpB*, Δ*ctpD*	This study
S1838	Tet-On::*cre*, Δ*oahA*, Δ*ctpA-F/amyA*::CT*ctpA*	This study
S1836	Tet-On::*cre*, Δ*oahA*, Δ*ctpA-F/amyA*::CT*ctpB*	This study
S1840	Tet-On::*cre*, Δ*oahA*, Δ*ctpA-F/amyA*::CT*ctpC*	This study
S1842	Tet-On::*cre*, Δ*oahA*, Δ*ctpA-F/amyA*::CT*ctpD*	This study
S1844	Tet-On::*cre*, Δ*oahA*, Δ*ctpA-F/amyA*::CT*ctpE*	This study
S1846	Tet-On::*cre*, Δ*oahA*, Δ*ctpA-F/amyA*::CT*ctpF*	This study
S1850	Tet-On::*cre*, Δ*oahA,* OE*ctpA*	This study
S1848	Tet-On::*cre*, Δ*oahA,* OE*ctpB*	This study
S1852	Tet-On::*cre*, Δ*oahA,* OE*ctpD*	This study
Plasmids		
pLH331	*lox*P*-hph-lox*P, *hyg^r^, kan^r^*	Xu et al. (2019)
pLH594	*lox*P*-hph-lox*P*, hyg^r^, ppt^r^, kan^r^*	This study
pLH605	*lox*P*-hph-lox*P*, hyg^r^, ppt^r^, kan^r^,* Δ*ctpA*	This study
pLH634	*lox*P*-hph-lox*P*, hyg^r^, ppt^r^, kan^r^,*Δ*ctpB*	This study
pLH639	*lox*P*-hph-lox*P*, hyg^r^, ppt^r^, kan^r^,*Δ*ctpC*	This study
pLH637	*lox*P*-hph-lox*P*, hyg^r^, ppt^r^, kan^r^,*Δ*ctpD*	This study
pLH644	*lox*P*-hph-lox*P*, hyg^r^, ppt^r^, kan^r^,* Δ*ctpE*	This study
pLH666	*lox*P*-hph-lox*P*, hyg^r^, ppt^r^, kan^r^,*Δ*ctpF*	This study
pLH924	*lox*P*-hph-lox*P*, hyg^r^, ppt^r^, kan^r^,* Δ*amyA*	This study
pLH926	*lox*P*-hph-lox*P*, hyg^r^, ppt^r^, kan^r^,* Δ*amyA, CTctpA*	This study
pLH925	*lox*P*-hph-lox*P*, hyg^r^, ppt^r^, kan^r^,* Δ*amyA, CTctpB*	This study
pLH927	*lox*P*-hph-lox*P*, hyg^r^, ppt^r^, kan^r^,* Δ*amyA, CTctpC*	This study
pLH928	*lox*P*-hph-lox*P*, hyg^r^, ppt^r^, kan^r^,* Δ*amyA, CTctpD*	This study
pLH974	*lox*P*-hph-lox*P*, hyg^r^, ppt^r^, kan^r^,* Δ*amyA, CTctpE*	This study
pLH975	*lox*P*-hph-lox*P*, hyg^r^, ppt^r^, kan^r^,* Δ*amyA, CTctpF*	This study
pLH1038	*lox*P*-hph-lox*P, *hyg^r^, kan^r^,* P*gpdA*::*ctpA*	This study
pLH1037	*lox*P*-hph-lox*P, *hyg^r^, kan^r^,* P*gpdA*::*ctpB*	This study
pLH1039	*lox*P*-hph-lox*P, *hyg^r^, kan^r^,* P*gpdA*::*ctpD*	This study

*hyg^r^*: hygromycin B resistance; *ppt^r^*: phosphinothricin resistance; *kan^r^*: kanamycin resistance; OE: overexpression; CT: complementation.

### Mining of putative mitochondrial citrate transporters (CTPs) in the *Aspergillus niger* genome

The *A. niger* 20131226 filtered model-protein database (*A. niger* ATCC 1015 v4.0)^1^ was searched with *Sc*Ctp1 protein sequence from *S. cerevisiae* S288c (accession no. NP_009850) through online blastp alignment program. With reference to genome annotation and the fact that shuttle transport of citric acid and dicarboxylic acid, sequences annotated as tri- and di-carboxylate transporter were both candidates for further analysis. Domain identification, annotation, and architectures of the obtained hit sequences were performed using the web resource SMART version 9^2^ with the default parameters. Transmembrane domains were analyzed through the Phyre2 web portal.^3^ Proteins without the Mito_carr (PF00153) domain were eliminated (Table 2). Alignment of candidates was performed with DNAMAN software Version 10. A phylogenetic tree was also established with MEGA software Version 10.1.7 by neighbor-joining statistical method. Accession numbers of the sequences for phylogenetic-tree construction are listed in Supplementary Table 2.

**Table 2:** Characteristics of Ctps in *Aspergillus niger*.

Protein		Deduced polypeptide			Genomic locus
Name	Protein identifier	Length (aa)	MW (kDa)	TMD number	
CtpA	ASPNIDRAFT_136079	296	32.47	6	chr_7_1:2781021–2,782,333
CtpB	ASPNIDRAFT_42578	296	31.72	6	chr_8_1:20139–21,262
CtpC	ASPNIDRAFT_194825	325	35.04	6	chr_6_3:242730–243,940
CtpD	ASPNIDRAFT_52803	305	33.32	6	chr_8_2:299722–300,991
CtpE	ASPNIDRAFT_41991	310	33.58	6	chr_1_2:1641656–1,642,901
CtpF	ASPNIDRAFT_174907	310	33.55	6	chr_4_2:469206–470,397

### Genomic DNA, total RNA isolation, and transcription analyses under citric acid accumulation condition

For genomic DNA isolation, 1 × 10^8^ conidia were inoculated into 50 ml of potato dextrose broth for 24 h, and mycelia were harvested followed by washing with water twice. The washed mycelia were ground into powder in liquid nitrogen. The genomic DNA was extracted using DNA extraction buffer (100 mM NaCl, 50 mM EDTA, 50 mM Tris, and 1.0% SDS; pH 8.5). Proteins and RNA were eliminated with phenol buffer and RNaseA, respectively. For total RNA isolation, mycelia were collected under indicated conditions, and total RNA was isolated following a previously described method (Cao et al., 2020). cDNA was synthesized using PrimeScript™ II 1st-Strand cDNA Synthesis Kit (TaKaRa Biomedical Technology Co., Ltd., Beijing, China). Transcription levels of the putative *ctpA* to *ctpF* in *A. niger* were determined by RT-qPCR. Primers are listed in Supplementary Table 3.

### Plasmid construction

Plasmids pLH605, pLH634, pLH639, pLH637, pLH644, pLH666, and pLH924 for *ctpA* to *ctpF* and *amyA* (ASPNIDRAFT_47911) disruptions were constructed as previously described (Cao et al., 2020). In a typical procedure, the dual flanked sequences of *ctpA* coding region were amplified with P3424/3425 and P3426/3427 followed by ligation into the upstream and downstream of the hygromycin resistance cassette (*lox*P-*hph*-*lox*P) in pLH594, respectively, to obtain the *ctpA* disruption plasmid pLH605. *ctpB-ctpF* and *amyA* disruption plasmids were constructed following the same strategy.

To construct the *ctpA* complementation plasmid integrated at the *amyA* locus, a *ctpA* expression cassette with its native promoter and terminator was obtained with P3407/3408. The resultant fragment was digested with *Bam*H I/*Sac* I and ligated into pLH924 to obtain pLH926. The same strategy was used for the construction of *ctpB* to *ctpF* complement plasmids.

The plasmid pLH1038 for *ctpA* overexpression under the control of glyceraldehyde-3-phosphate dehydrogenase (*gpdA*) promoter was constructed as follows. P3619/3620 was used to amplify the cDNA of *ctpA*, and the obtained fragment was digested with *Bam*H I/*Eco*R I, followed by ligation into pLH454 to obtain pLH1038. *ctpB* and *ctpD* gene overexpression plasmids were constructed following the same strategy. Primers used are listed in Supplementary Table 3.

### Transformation of *Aspergillus niger* for disruption, individual reintroduction of *ctpA* to *ctpF*, and overexpression of *ctpA*, *ctpB*, and *ctpD*

*Agrobacterium*-mediated transformation was applied for *A. niger* genetic manipulation as previously described (Cao et al., 2020). *ctpA* disruptants were obtained by introducing pLH605 into *A. niger* S422. Transformants were selected on CM plates supplemented with cefotax thiazide sodium (0.10 g/l), hygromycin B (0.25 g/l), ampicillin (0.10 g/l), and streptomycin (0.10 g/l) at 28°C for 5 days, followed by screening on PDA plates with hygromycin B (0.25 g/l) and MM with glufosinate ammonium (1.00 g/l), respectively. According to the principle of homologous recombination, Δ*ctpA* candidates that were hygromycin B resistant and glufosinate ammonium sensitive were identified by PCR analyses with P3416/P3417, P3416/P641, P642/P3419, P3418/P3419, and P3416/P3419. The resultant *ctpA* deficiency mutant was named S838 (Supplementary Figure 3A). In the same way, P1967/P1968, P1967/P641, P642/P1970, P1969/P1970, and P1967/P1970 were used for Δ*ctpB* candidate identification; P2009/P2010, P2009/P641, P642/P2012, P2011/P2012, and P2009/P2012 were used for Δ*ctpC* candidate identification; P2013/P2014, P2013P641, P642/P2016, P2015/P2016, and P2013/P2016 were used for Δ*ctpD* candidate identification; P2017/P2018, P2017/P641, P642/P2020, P2019/P2020, and P2017/P2020 were used for Δ*ctpE* candidate identification; and P2021/P2022, P2021/P641, P642/P2024, P2023/P2024, and P2021/P2024 were used for Δ*ctpF* candidate identification, respectively. Individual deficiency mutants obtained for *ctpB*, *ctpC*, *ctpD*, *ctpE*, and *ctpF* were named S836, S1152, S865, S866, and S938 (Supplementary Figures 3B–F). Deletion mutant for all six *ctp*s was also obtained by using the Cre-*lox*P-based genetic system (Xu et al., 2019) and named S1696 (Δ*ctpA-F*). The primers used for PCR verification are listed in Supplementary Table 3.

To determine the role of CtpA in citric acid accumulation, pLH926 harboring *ctpA* expression cassette with its native promoter was reintroduced into the *amyA* locus in *A. niger* S1696 (Δ*ctpA-F*). The obtained *ctpA* complementation strain was named S1838. Other *ctp*s were also individually reintroduced into *A. niger* S1696 (Δ*ctpA-F*), and the resultant strains were named S1836, S1840, S1842, S1844, and S1846, respectively.

### Growth assay and conidia formation of *Aspergillus niger* parent and mutant strains

To investigate the growth differences, 5 × 10^4^ conidia of *A. niger* parent and the indicated mutant strains in distilled water were inoculated on PDA plates at 28°C for 4 days. Morphology and conidia formation were photographed and analyzed. To establish the growth curve, 1 × 10^6^ conidia of *A. niger* were inoculated into 20 ml of citric acid fermentation medium. At the indicated time, the culture broths were passed through qualitative filter paper (Whatman No.4), and trapped mycelia were completely dried at 105°C.

### Shake-flask fermentation for assaying citric acid production

To assess the citric acid accumulations, 2 × 10^6^ conidia/ml of *A. niger* mutants were inoculated into 50 ml of citric acid fermentation medium (10% sucrose, 0.25% NH_4_NO_3_, 0.1% MgSO_4_·7H_2_O, 0.1% KH_2_PO_4_, and yeast extract 0.05%; pH 2.5) in 250 ml Erlenmeyer flasks at 28°C and 200 rpm for 5 days. Fermentation broths were sampled at indicated time points for organic acid analyses as previously described (Cao et al., 2020).

### Intracellular acetyl-CoA concentration measurement

A total of 2 × 10^6^ conidia/mL for each *A. niger* strain were inoculated into 50 ml of citric acid fermentation medium in 250 ml Erlenmeyer flasks at 28°C, and 200 rpm for 3 days. The obtained mycelia were washed and ground into powder in the presence of liquid nitrogen. The acetyl-CoA concentration was measured with an Acetyl-CoA Assay Kit (Solarbio, BC0980), and the total protein concentration was determined using a BCA Protein Assay Kit (TaKaRa, T9300A) according to the manufacturer’s instructions. Optical density at 340 nm and 562 nm was measured for Acetyl-CoA and protein contents quantification, respectively, with a UV-3600i Plus system (Shimadzu, Japan).

### Statistical analyses

All experiments were performed in triplicate, and the mean values were compared using two-tailed Student’s *t*-test. ^*^p < 0.05, ^**^p < 0.001, and ^****^p < 0.0001 were considered statistically significant.

## Results

### Identification of mitochondrial citrate transporters in *Aspergillus niger* (Ctps)

Previous studies have demonstrated that a protein CtpA was shown to play an important role in citrate transport across mitochondria membrane and citric acid production in *A. niger* WU-2223 l and *A. kawachii* SO2 (Kirimura et al., 2016; Kadooka et al., 2019). A Preliminary experiments showed that deletion of *ctpA* in *A. niger* strain S422 resulted in the production of citric acid decreased by 15.8 and 18.3% at 3 and 5 days, respectively (Supplementary Figure 1). This suggested that CtpA was indeed involved in the transport of citrate across the mitochondrial membrane, and other undiscovered Ctps might were also involved.

**Figure 1:**
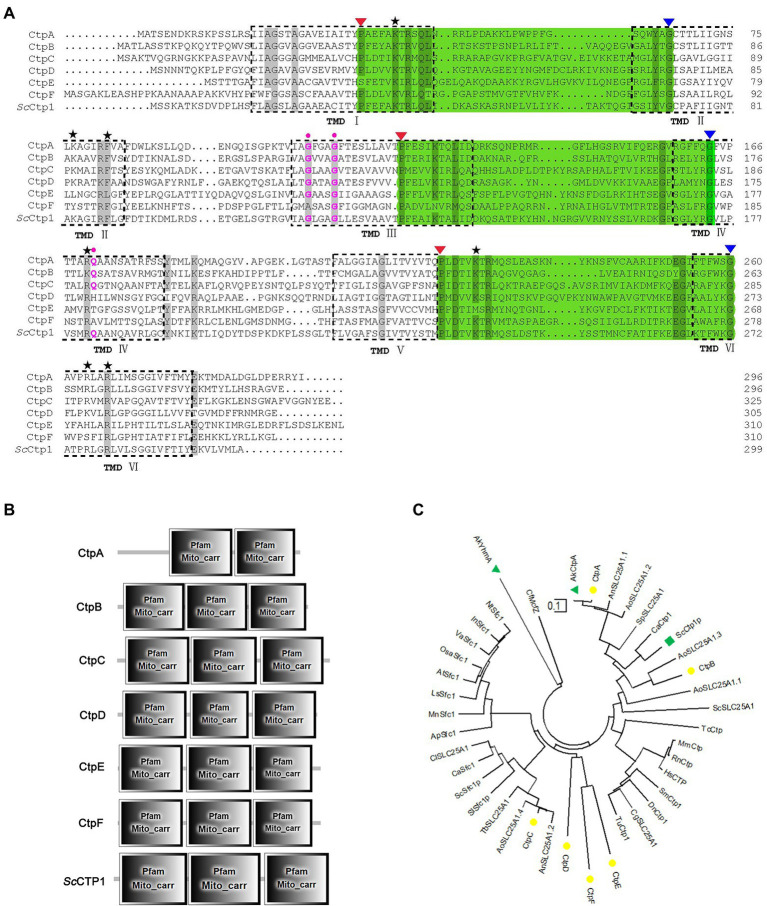
Sequence features and domain analyses of CtpA to CtpF. **(A)** Multiple sequence alignment of CtpA to CtpF amino acid sequence against their yeast ortholog *Sc*Ctp1. Six predicted transmembrane domains (TMD) based on *Sc*Ctp1 are marked as TMD I to VI. Note: three symbolic motifs PX[D/E]XX[K/R]X[K/R] (20–30 residues) [D/E]GXXXX[W/Y/F][K/R]G are in bottle-green background, and the conservative P and G are marked with red and blue inverse triangles, respectively; black Pentastar, citrate binding sites; pink dots, amino acid residues for dimer interface. **(B)** CtpA to CtpF and *Sc*Ctp1 domain architecture. **(C)** Neighbor-joining phylogenetic tree established using MEGA software Version 10.1.7. CtpA to CtpF are marked with yellow solid circle, *Sc*Ctp1 is marked with green solid triangle, and *Ak*Ctp and *Ak*YhmA are marked with green square.

Here, *Sc*Ctp1 from *S. cerevisiae* is an ortholog of human SLC25A1 located in the inner mitochondrial membrane and is responsible for the citrate-malate shuttles (Kaplan et al., 1995; Gnoni et al., 2009; Palmieri, 2013). In the present study, using *Sc*Ctp1 as query sequence, 23 sequences were obtained (Supplementary Table 1). After sequence annotation screening and functional domain analysis, six putative Ctps were identified in *A. niger* ATCC 1015 model-protein database, namely CtpA to CtpF (Table 1). It should be noted that, based on the fact that the antiport of citrate and dicarboxylate (malate, oxoglutarate, oxaloacetate, etc) is the main manner of citrate transport from mitochondria to cytoplasm (Karaffa and Kubicek, 2003; Kirimura et al., 2016, 2019; Kadooka et al., 2019; Yang et al., 2019), the annotated putative dicarboxylate transporters were also named citrate transporters here. Among them, only CtpA has been investigated in *A. niger* WU-2223 l strain and *A. luchuensis* mut. *Kawachii* (Kirimura et al., 2016; Kadooka et al., 2019). Alignment with *Sc*Ctp1 showed that CtpA to CtpF exhibited 45.66, 42.9, 29.85, 23.89, 21.05, and 20.38% amino acid sequence identities, respectively. Six transmembrane domains (TMD I–VI; Figure 1A and Supplementary Figure 2) and three conserved motifs were found in each putative Ctp (Figure 1A), which were typical characteristics of all mitochondrial transporter family members (Palmieri, 2013). The predicted amino acid residues for dimer formation and citrate binding can also be found in most sequences and marked with different color symbols (Figure 1A; Kirimura et al., 2016; Kadooka et al., 2019). Functional domain analyses revealed that CtpA had two mito_carr domains, whereas other Ctps had three mito_carr domains in a tandem manner (Figure 1B). These analyses indicate that CtpA to CtpF might have citrate-transport activity. Phylogenetic analysis showed that CtpA, CtpB, *Ak*CtpA, and *Sc*Ctp1 belong to one evolutionary branch, whereas CtpC, CtpD, CtpE, and CtpF are assigned to another common evolutionary branch (Figure 1C). It should be noted that although *Ak*YhmA was reported to be a mitochondrial citrate transport protein (Kadooka et al., 2019), it had a relative distant evolutionary relationship with the putative Ctps (Figure 1C).

### Transcription analyses of *ctpA* to *ctpF*

**Figure 2:**
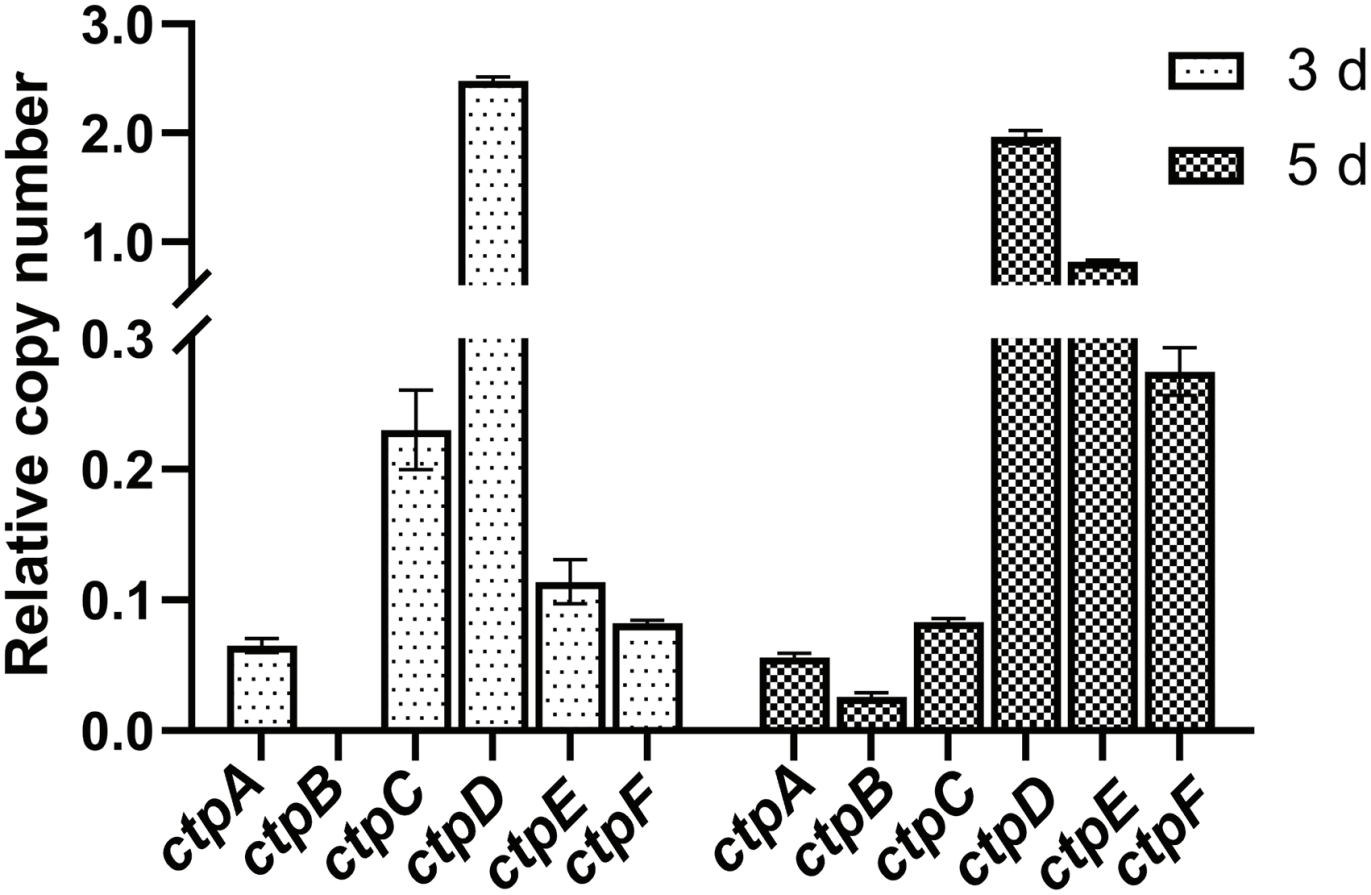
RT-qPCR analysis of *ctpA* to *ctpF* relative expression in S422 under citric acid fermentation conditions at 28°C, and 200 rpm for 3 days and 5 days, respectively. All expression values were normalized to the expression level of *actA* (ASPNIDRAFT_200483).

To determine the expression profiles of the putative *ctpA* to *ctpF* during citric acid accumulation, the total RNA of the parent strain *A. niger* S422 cultivated in citric acid fermentation medium for 3 and 5 d was, respectively, extracted for RT-qPCR analyses. As shown in Figure 2, except for *ctpB* that was transcribed only in the samples of 5-d fermentation, *ctpA*, *ctpC*, *ctpD*, *ctpE*, and *ctpF* were transcribed in the samples of both 3- and 5-day fermentation. Notably, in *A. niger* WU-2223 l, no transcription of *ctpB* was detected in SLZ medium (Kirimura et al., 2016). Citric acid fermentation medium in this study and SLZ medium are different in carbon source and mineral salt, which may lead to different expression profiles of *ctpB*.

### Phenotype analyses of *A. niger* mutants with individual *ctp* disruption or deletions of all six *ctp*s

**Figure 3:**
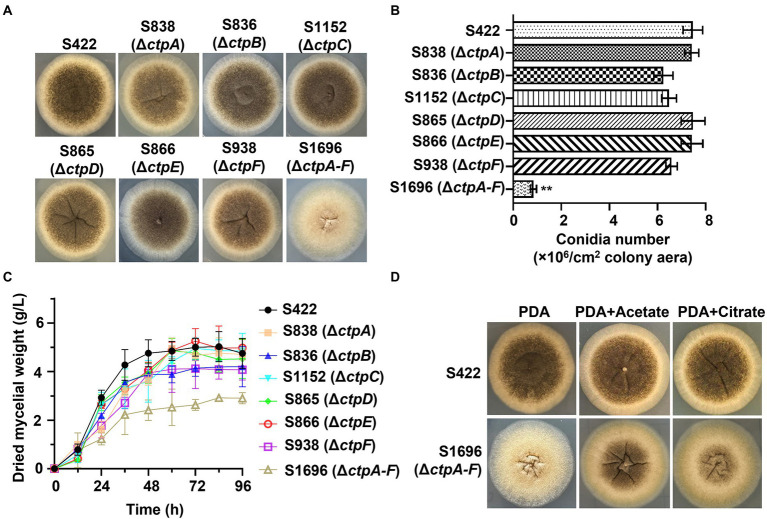
Phenotype analyses of *ctp* disruptions strains. **(A)** Morphology of *A. niger* S422, individual *ctp* disruption mutants, and Δ*ctpA-F* mutant grown on PDA plate at 28°C for 4 d. **(B)** The number of conidia per square centimeter of the indicated strains grown on PDA plates at 28°C for 4 d. **(C)** Growth curves of *A. niger* S422 and the indicated mutants in 50 ml of citric acid fermentation medium at 28°C, and 200 rpm. **(D)** Morphology of *A. niger* S422 and S1696 (Δ*ctpA-F*) grown on PDA plate or supplemented with 5 mM acetate or 10 mM citrate.

**Figure 4:**
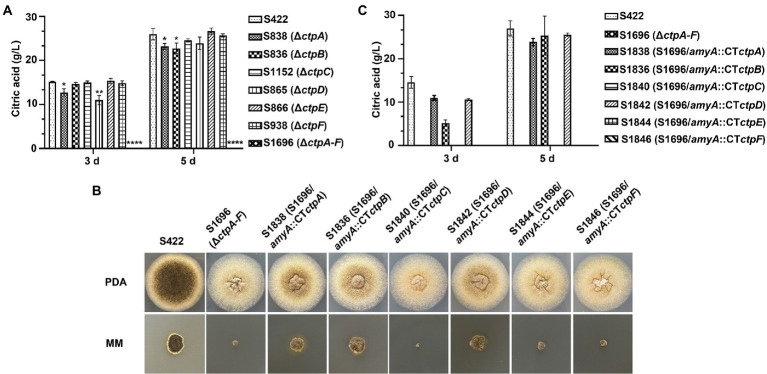
Citric acid production and morphology analyses of *A. niger* strains. **(A)** In a 250 ml shake flask, 1 × 10^8^ conidia of each *A. niger* strain were inoculated in 50 ml of citric acid fermentation medium at 28°C for 3 and 5 days, respectively. Citric acid titers were determined by HPLC. **(B)** Morphology of *A. niger* S422 and S1696 (Δ*ctpA-F*) and each Ctp reintroduction strain grown on PDA or MM agar plates at 28°C for 4 days. **(C)** Citric acid titers of each Ctp reintroduction *A. niger* strains were determined.

To investigate the physiological effects of CtpA to CtpF on *A. niger* growth and conidia formation, mutants with disruption of individual *ctp*s or deletion of all six *ctp*s were constructed (Supplementary Figures 3A–F). Compared with the parent strain S422 on PDA plates, all the individual *ctp* disruption mutants exhibited similar colonial morphologies, whereas S1696 (Δ*ctpA-F*) colony became more fluffy and albino, and the conidia formation was significantly impaired (Figure 3A). Additionally, S1696 (Δ*ctpA-F*) formed a smaller colony than the control strain S422 on MM plates (Figure 4B). Furthermore, the conidia formation of individual mutants was measured after their growth on PDA plates for 5 days and results showed that the number of conidia per square centimeter for individual *ctp* mutants decreased to varying degrees but was not statistically significant, whereas the number of conidia formed by S1696 (Δ*ctpA-F*) was reduced to only 11.5% of that produced by the parent strain S422 (Figure 3B). Under citric acid fermentation condition, each *ctp* disruptant had a slightly slow growth rate, whereas S1696 (Δ*ctpA-F*) exhibited a significantly reduced growth rate than S422 (Figure 3C). Similarly, previous studies also reported that *ctpA* disruption caused growth delay in *A. niger* strain WU-2223l and *A. kawachii* strain SO2 (Kirimura et al., 2016; Kadooka et al., 2019). Citrate transporters are considered to export citrate from mitochondria to cytoplasm and, in turn, citrate is cleaved to supply acetyl-CoA. In the cytoplasm, citrate participates in fatty acid and cholesterol biosynthesis and regulates acetyl-CoA carboxylase activity (Gnoni et al., 2009; Chen et al., 2014; Kadooka et al., 2019; Ruprecht and Kunji, 2020). Accordingly, the complete deficiency of *ctp*s is predicted to result in the blockage of citrate transport, which, in turn, affected the supply of acetyl-CoA in the cytoplasm and caused growth defects. To test this hypothesis, the effects of exogenous addition of citrate or acetate on the growth defect of S1696 (Δ*ctpA-F*) were investigated. As shown in Figure 3D, defects of pigment production and conidia formation in S1696 (Δ*ctpA-F*) mutant were partially relieved with the addition of acetate or citrate. These results further demonstrated that disturbance of citrate transport caused by *ctp* deletion affected growth and conidia formation.

### Effects of putative ctps on citric acid accumulation

To investigate the contribution of Ctp to citric acid production in *A. niger*, citric acid titers of individual *ctp* disruptants and S1696 (Δ*ctpA-F*) were determined. As shown in Figure 4A, in the initial 3 days fermentation, S836 (Δ*ctpB*), S1152 (Δ*ctpC*), S866 (Δ*ctpE*), and S938 (Δ*ctpF*) strains had similar citric acid titers to the parent strain S422, whereas in S838 (Δ*ctpA*) and S865 (Δ*ctpD*) fermentation broth, the titers of citric acid decreased by 15.8 and 27.4%, respectively. On the 5 days of the late fermentation stage, S1152 (Δ*ctpC*), S865 (Δ*ctpD*), S866 (Δ*ctpE*), and S938 (Δ*ctpF*) strains had comparable levels of citric acid, whereas S838 (Δ*ctpA*) and S836 (Δ*ctpB*) exhibited a significant decrease in citric acid titers (18.3, 12.3%, respectively). To further investigate their roles in citric acid accumulation, mutants with double deletions of *ctpA*, *ctpB*, and *ctpD* were constructed (Supplementary Figures 3M–O). Although citric acid titers of mutants S2422 (Δ*ctpA*, Δ*ctpB*), S1920 (Δ*ctpA* and Δ*ctpD*), and S1995 (Δ*ctpB* and Δ*ctpD*) also declined by 16.6, 38.7 and 36.8% at 3 days, and 37.9, 21.1 and 16.9% at 5 days, respectively, S2152 (Δ*ctpA*, Δ*ctpB*, and Δ*ctpD*) showed decreases of 89.9 and 91.0% on the 3 days and 5 days, respectively (Supplementary Figure 4). Similar to S2152 (Δ*ctpA*, Δ*ctpB*, and Δ*ctpD*), deletions of all the six putative Ctps in S1696 resulted in no detectable levels of citric acid accumulation in the fermentation broth on the 3 days or 5 days of fermentation (Figure 4A). These results indicate that CtpA, CtpB, and CtpD are responsible for citrate export from mitochondria to cytoplasm at different fermentation stages in *A. niger*. In other words, CtpB may contribute in the late stage of fermentation, CtpD may primarily function in the early stage, and CtpA may play an important role throughout the entire fermentation stage. The effects of CtpA, CtpB, and CtpD on citric acid accumulation were also consistent with their expression profiles during the fermentation process (Figure 2).

To further determine whether *ctpA* to *ctpF* can restore citric acid production in S1696 (Δ*ctpA-F*), *ctpA* to *ctpF* expression cassettes were individually introduced with their respective native promoters at the *amyA* locus (Supplementary Figures 3G–I) and all obtained strains were grown on PDA and MM agar plates, respectively. As shown in Figure 4B, defects of conidia formation, pigment production, and growth were partially suppressed by the reintroduction of *ctpA*, *ctpB*, or *ctpD*. The shake-flask fermentation test also showed that the citric acid titers of S1838 (S1696/*amyA*::*ctpA*), S1836 (S1696/*amyA*::*ctpB*), and S1842 (S1696/*amyA*::*ctpD*) recovered to the levels of 75.3, 35.4, and 72.6% at 3 days fermentation, as well as 93.8, 88.5, and 94.6% at 5 days, respectively, of the parent strain S422. These results indicate that CtpA, CtpB, and CtpD participate in citrate transport across the mitochondrial membrane in a redundant manner.

### Effects of *ctpA*, *ctpB*, or *ctpD* reintroduction on intracellular acetyl-CoA levels

**Figure 5:**
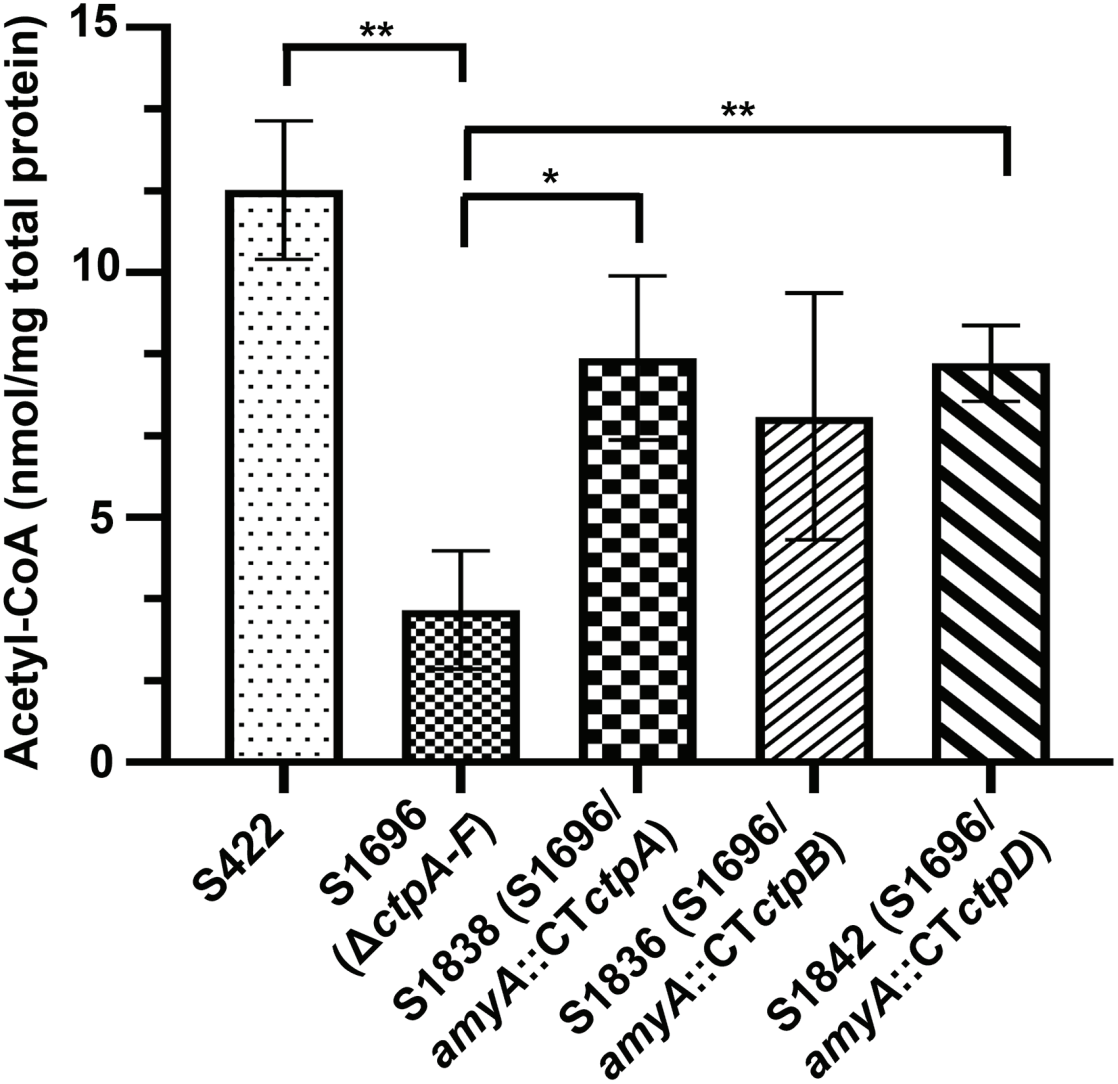
Intracellular acetyl-CoA concentrations of the indicated *A. niger* strains. About 1 × 10^8^ conidia of each indicated *A. niger* strain were inoculated in 50 ml of citric acid fermentation medium at 28°C for 3 days, and the intracellular acetyl-CoA concentrations were determined. *p < 0.05, **p < 0.001, and ****p < 0.0001 were considered statistically significant. This meaning are described in “Statistical analyses” section.

Citrate in the cytoplasm is cleaved by ATP-citrate lyase to supply acetyl-CoA for energy and biomolecule biogenesis, as well as protein acetylation. Thus, its sufficient supply is essential for normal growth and development (Chypre et al., 2012; Chen et al., 2014). Meanwhile, the addition of acetate and citrate partially recovered the conidia formation (Figure 3D) which may be due to the production of acetyl-CoA from acetate or citrate supplement. Based on the above results and prediction, the intracellular variations of acetyl-CoA with *ctpA*, *ctpB*, or *ctpD* reintroduction were further examined. As illustrated in Figure 5, loss of all *ctps* resulted in decrease in intracellular acetyl-CoA concentrations by 73.4%, whereas *ctpA* reintroduction into S1696 (Δ*ctpA-F*) restored the acetyl-CoA concentrations from 3.11 nmol/mg to 8.26 nmol/mg, and *ctpB* or *ctpD* reintroduction elevated the acetyl-CoA concentrations to 7.06 nmol/mg and 8.14 nmol/mg, respectively, than that in S1696 (Δ*ctpA-F*). These findings suggested that CtpA, CtpB, and CtpD were involved in citrate transport from mitochondria to cytoplasm and in turn affected acetyl-CoA biosynthesis. Moreover, the complete blockage of this process led to growth and development defects as displayed by strain S1696 (Δ*ctpA-F*).

### Overexpression of respective *ctpA*, *ctpB*, or *ctpD* elevated citric acid accumulation

**Figure 6:**
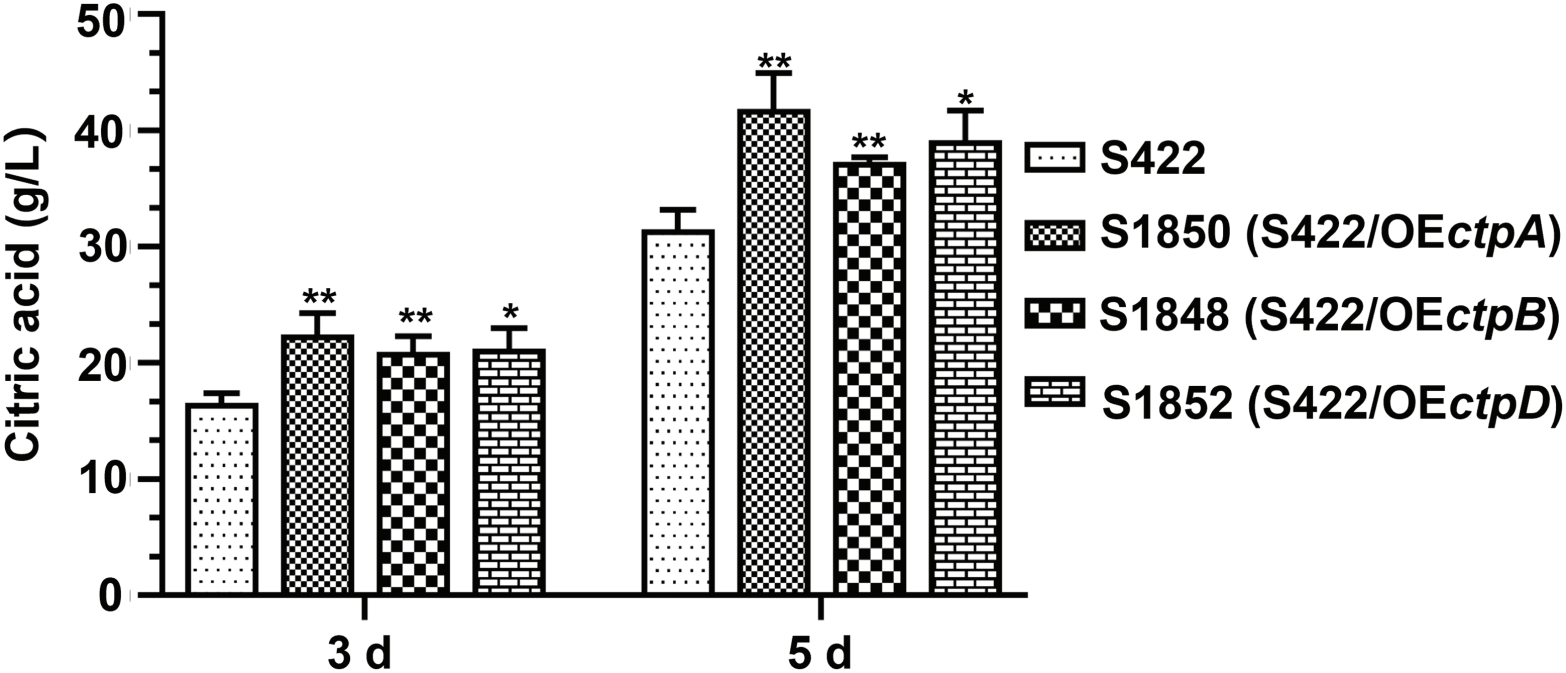
Citric acid production by *A. niger* S422 and S1848 (S422/OE*ctpB*), S1850 (S422/OE*ctpA*), and S1852 (S422/OE*ctpD*). About 1 × 10^8^ conidia of *A. niger* were inoculated in 50 ml of citric acid fermentation medium at 28°C for 3 and 5 days, respectively. Citric acid titers were determined by HPLC. *p < 0.05, **p < 0.001, and ****p < 0.0001 were considered statistically significant. This meaning are described in “Statistical analyses” section.

Given that CtpA, CtpB, and CtpD were shown to participate in citric acid transport across the mitochondrial inner membrane and affected citric acid accumulation, they were reasonable targets for engineering more efficient strains for citric acid production. Therefore, *ctpA*, *ctpB*, or *ctpD* overexpression cassettes driven by the constitutive promoter P*gpdA* were individually introduced into the parent strain S422. The overexpression transformants of *ctpA*, *ctpB*, or *ctpD*, namely, S1850 (S422/OE*ctpA*), S1848 (S422/OE*ctpB*) and S1852 (S422/OE*ctpD*), were obtained and applied for shake-flask citrate fermentation. As shown in Figure 6, compared with S422, the respective overexpressions of *ctpA*, *ctpB*, and *ctpD* caused 32.8, 19.3, and 24.2% increase in citric acid accumulation at 5 days, respectively. The process of citrate export from mitochondria to cytoplasm has been predicted to be one of the bottlenecks in citrate production (Torres, 1994a,b), and enhancement of this process by the overexpression of these newly identified Ctps in the present study confirmed this prediction. Therefore, multiple Ctps are involved in citrate export from mitochondria to cytoplasm and enhancement of this process boosts extracellular citric acid accumulation.

## Discussion

Citric acid, primarily produced by the submerged aerobic fermentation of *A. niger*, is the largest consumed bulk organic acid and is extensively used in food, medicine, detergent, and cosmetics (Behera, 2020). Studies on improving the production process of citric acid fermentation are extensive. As the basis of fermentation, improvements in the citric acid-producing *A. niger* strain has important practical significance for promoting the development of the citric acid industry. In particular, most studies focus on engineering glycolysis and the TCA pathway for enhanced citric acid biosynthesis, elimination of inhibitor effects on citric acid biosynthesis and by-product formation, and enhancement in the supplement pathway (Yin et al., 2015). Additionally, citric acid transport processes are potential modification targets for enhancing its accumulation (Karaffa and Kubicek, 2003; Kadooka et al., 2019; Odoni et al., 2019; Steiger et al., 2019; Nakamura et al., 2020).

Here, the genes encoding mitochondrial citrate transporters responsible for citrate transport from mitochondria to cytoplasm were systematically mined and identified in *A. niger* genome. Six candidates, namely, CtpA to CtpF, were identified based on sequence homology with *Sc*Ctp1 from *S. cerevisiae*. All six Ctps exhibited similar domain-organization patterns and conserved functional amino acid residues but belong to different evolutionary branches (Figure 1C). A previous study on *A. niger* WU-2223l has shown that *ctpB* is not expressed under the conditions examined (Kirimura et al., 2016). However, under our citric acid fermentation conditions, *ctpB* transcripts were detected on the 5 days of fermentation (Figure 2), indicating that CtpB may be an important contributor of citric acid transport across the mitochondrial inner membrane in the late stage of citric acid fermentation. Other *ctp*s were expressed during citric acid fermentation, suggesting that they may play different roles in citrate export in different fermentation stages. Although individual *ctp* disruptants exhibited similar growth and conidia formation to the parent strain, disruption of all six *ctp*s led to growth defects, diminished pigmentogenesis, and diminished conidia formation (Figures 3A,B, 4B). Citrate supplement or introduction of respective CtpA/CtpB/CtpD also partially relieved the growth and development defects (Figures 3D, 4B). Additionally, no changes in temperature-dependent growth were observed, as previously reported in *A. niger* WU-2223 l and *A. kawachii* (data not shown; Kirimura et al., 2016; Kadooka et al., 2019). Ctps were also found to be involved in acetyl-CoA synthesis regulation (Figure 5). Ctps are considered to be responsible for the supply of cytosolic citrate to generate acetyl-CoA for cell growth and development (Mysyakina and Feofilova, 2011; Palmieri, 2013; Chen et al., 2014; Kadooka et al., 2019). The defects in growth and development in S1696 (Δ*ctpA-F*) may be partially caused by the insufficient supply of citrate in the cytosol.

A previous study on *A. niger* WU-2223l found that CtpA participates in citric acid accumulation in the early-log phase of fermentation. In addition to CtpA, other Ctps were suggested to be involved in citric acid production in *A. niger* (Kirimura et al., 2016). In *A. kawachii*, two mitochondrial citrate transporters, *Ak*CtpA (AKAW_03754) and *Ak*YhmA (AKAW_06280), were shown to be involved in citric acid accumulation and required for acetyl-CoA biosynthesis (Kadooka et al., 2019). In the present study, disruption of individual *ctpA*, *ctpB*, or *ctpD* led to significantly decreased accumulation of extracellular citric acid (Figure 4A), and their respective reintroduction to S1696 (Δ*ctpA-F*) partially restored the citric acid accumulation (Figure 4C). Importantly, overexpression of individual *ctpA*, *ctpB*, or *ctpD* under the constitutive promoter P*gpdA* caused significant increases in citric acid titers (Figure 6). These findings confirmed that CtpA, CtpB, and CtpD are major contributors to citrate transport in *A. niger* and function in a redundant manner. Although through blastp alignment program, one sequence ASPNIDRAFT_136079 (*An*YhmA) was found to be a homolog of *Ak*YhmA in *A. niger*, CtpA and *An*YhmA exhibited only 18.87% sequence identity. Based on the results that citric acid accumulation was almost abolished when *ctpA*, *ctpB,* and *ctpD* were co-deleted (Supplementary Figure 4), *An*YhmA might not participate in citrate transport across mitochondria in *A. niger* S422. Mitochondrial citrate transporters are considered to participate in citrate export, and Ctps such as *Sc*Ctp1, *Rn*Ctp, and *Ak*CtpA were previously shown to exhibit different substrate specificities by reconstitution using liposomes. However, the molecular details of Ctps in *A. niger* are not completely understood and require further studies. Notably, although CtpE and CtpF share an evolutionary branch with the previously reported *Ak*CtpA and *Ak*YhmA, our data failed to support their independent involvement in extracelluar citric acid accumulation, similar to the case of CtpC. Further studies are needed to clarify the gene functions of *ctpC*, *ctpE*, and *ctpF*.

In summary, three mitochondrial citrate transporters, namely, CtpA, CtpB, and CtpD, were identified to be involved in citrate export from mitochondria to cytoplasm, thus contributing to extracellular citric acid accumulation. Additionally, given that citrate in the cytoplasm was the main substrate for acetyl-CoA synthesis, it is no surprising that CtpA, CtpB, and CtpD play important roles in strain growth, pigmentogenesis, and conidia formation. All these findings increased the understanding of the mechanisms of citrate transport and extracellular accumulation, thus providing fundamental knowledge for engineering more efficient commercial strains of *A. niger* for citrate production.

## Data availability statement

The original contributions presented in the study are included in the article/Supplementary material, further inquiries can be directed to the corresponding author.

## Author contributions

WC analyzed the data and wrote the manuscript. LZ and LW generated all genetic constructs. MZ and JL performed the shake-flask fermentation experiments. JL and ZX contributed to scientific discussions and commented on the manuscript. HL supervised the work and revised the manuscript. All authors contributed to the article and approved the submitted version.

## Funding

This study was supported by the National Key Research and Development Program of China (2021YFC2100700) and the Tianjin Synthetic Biotechnology Innovation Capacity Improvement Project (TSBICIP-KJGG-006).

## Conflict of interest

The authors declare that the research was conducted in the absence of any commercial or financial relationships that could be construed as a potential conflict of interest.

## Publisher’s note

All claims expressed in this article are solely those of the authors and do not necessarily represent those of their affiliated organizations, or those of the publisher, the editors and the reviewers. Any product that may be evaluated in this article, or claim that may be made by its manufacturer, is not guaranteed or endorsed by the publisher.

## Footnotes

^1^ https://mycocosm.jgi.doe.gov/pages/blast-query.jsf?db=Aspni7

^2^ http://smart.embl-heidelberg.de/

^3^ http://www.sbg.bio.ic.ac.uk/phyre2/html/page.cgi?id=index
