# Supplementary material for: Identification and genetic characterization of mitochondrial citrate transporters in Aspergillus niger
Source: Front Microbiol. 2022 Sep 13;13:1009491. doi: 10.3389/fmicb.2022.1009491 (PMC9512666; doi:10.3389/fmicb.2022.1009491)
Supplement: Supplementary file 1 [file Data_Sheet_1.pdf]

Title:

Identification and genetic characterization of the mitochondrial citrate transporters in *Aspergillus niger*

Journal Name:

Frontiers in Microbiology

Authors:

Wei Cao[1, 2, 3], Licheng Zhang[1], Liu Wu[1], Mingyi Zhang[1], Jiao Liu[1, 2], Zhoujie Xie[1, 2], Hao Liu[1, 2, 3*]

Affiliation:

[1]MOE Key Laboratory of Industrial Fermentation Microbiology, College of Biotechnology, Tianjin University of Science and Technology, 300457, Tianjin, P. R. China

[2]Tianjin Engineering Research Center of Microbial Metabolism and Fermentation Process Control, Tianjin University of Science and Technology, 300457, Tianjin, P. R. China

[3]National Technology Innovation Center of Synthetic Biology, 300308, Tianjin, P. R. China

* Corresponding Author:

Phone: (86)-22-60600810. Fax: (86)-22-60602298. Email: liuhao@tust.edu.cn

Supplementary Table 1. Sequences obtained from alignments of *Sc*Ctp1 in *A. niger* ATCC 1015

NO.		Hit Name	locus_tag	Definition	K number	Score	EValue	Organism	Dataset
1	CtpA	Aspni7|1178940	ASPNIDRAFT_136079	An11g11230; hypothetical protein; solute carrier family 25 (mitochondrial citrate transporter), member 1	K15100	733	6E-70	Aspergillus niger ATCC 1015 v4.0	Aspni7_GeneCatalog_proteins_20131226.aa
2	CtpB	Aspni7|1178960	ASPNIDRAFT_42578	An18g00070; tricarboxylate transport protein; solute carrier family 25 (mitochondrial citrate transporter), member 1	K15100	681	3E-67	Aspergillus niger ATCC 1015 v4.0	Aspni7_GeneCatalog_proteins_20131226.aa
3	CtpC	Aspni7|1222809	ASPNIDRAFT_194825	An04g09030; succinate/fumarate transporter; solute carrier family 25 (mitochondrial citrate transporter), member 1	K15100	587	5E-40	Aspergillus niger ATCC 1015 v4.0	Aspni7_GeneCatalog_proteins_20131226.aa
4		Aspni7|1148364	ASPNIDRAFT_47290	An06g01730; RNA-splicing protein MRS3; solute carrier family 25 (mitochondrial iron transporter), member 28/37	K15113	368	3E-10	Aspergillus niger ATCC 1015 v4.0	Aspni7_GeneCatalog_proteins_20131226.aa
5		Aspni7|1104917	ASPNIDRAFT_209784	An07g03070; hypothetical protein; solute carrier family 25 (mitochondrial aspartate/glutamate transporter), member 12/13; contains three calcium-binding motifs Efh.	K15105	300	1E-17	Aspergillus niger ATCC 1015 v4.0	Aspni7_GeneCatalog_proteins_20131226.aa
6	CtpD	Aspni7|1113466	ASPNIDRAFT_52803	An08g01370; 2-oxodicarboxylate carrier 2; solute carrier family 25 (mitochondrial 2-oxodicarboxylate transporter), member 21	K15100	264	4E-14	Aspergillus niger ATCC 1015 v4.0	Aspni7_GeneCatalog_proteins_20131226.aa
7		Aspni7|1101715	ASPNIDRAFT_170858	An01g07650; solute carrier family 25, member 38	K15118	243	3E-11	Aspergillus niger ATCC 1015 v4.0	Aspni7_GeneCatalog_proteins_20131226.aa
8		Aspni7|1209512	ASPNIDRAFT_45765	An03g01110; carrier protein YMC1; solute carrier family 25 (mitochondrial	K15109	241	5E-15	Aspergillus niger ATCC 1015 v4.0	Aspni7_GeneCatalog_proteins_20131226.aa
9		Aspni7|1112876	ASPNIDRAFT_211899	An18g02830; WD repeat-containing protein 48	K15361	238	3E-08	Aspergillus niger ATCC 1015 v4.0	Aspni7_GeneCatalog_proteins_20131226.aa
10		Aspni7|1126790	ASPNIDRAFT_205206	solute carrier family 25 (mitochondrial carrier protein), member 16	K15084	237	4E-10	Aspergillus niger ATCC 1015 v4.0	Aspni7_GeneCatalog_proteins_20131226.aa
11		Aspni7|1163349	ASPNIDRAFT_36158	An01g04690; peroxisomal carrier protein		215	3E-07	Aspergillus niger ATCC 1015 v4.0	Aspni7_GeneCatalog_proteins_20131226.aa
12		Aspni7|1146086	ASPNIDRAFT_44373	An03g03360; carnitine/acyl carnitine carrier; solute carrier family 25 (mitochondrial carnitine/acylcarnitine transporter), member 20/29	K15109	213	2E-07	Aspergillus niger ATCC 1015 v4.0	Aspni7_GeneCatalog_proteins_20131226.aa
13		Aspni7|1180297	ASPNIDRAFT_126298	solute carrier family 25 (mitochondrial carnitine/acylcarnitine transporter), member 20/29	K15109	209	5E-07	Aspergillus niger ATCC 1015 v4.0	Aspni7_GeneCatalog_proteins_20131226.aa
14	CtpE	Aspni7|1141800	ASPNIDRAFT_41991	An14g06860; oxaloacetate transport protein	K15117	203	4E-10	Aspergillus niger ATCC 1015 v4.0	Aspni7_GeneCatalog_proteins_20131226.aa
15		Aspni7|1147568	ASPNIDRAFT_212044	An18g04220; ADP/ATP carrier protein; solute carrier family 25 (mitochondrial adenine nucleotide translocator), member 4/5/6/31	K05863	202	6E-10	Aspergillus niger ATCC 1015 v4.0	Aspni7_GeneCatalog_proteins_20131226.aa
16		Aspni7|1115499	ASPNIDRAFT_184977	An14g01860; hypothetical protein; solute carrier family 25, member 33/36	K15115	196	4E-09	Aspergillus niger ATCC 1015 v4.0	Aspni7_GeneCatalog_proteins_20131226.aa
17	CtpF	Aspni7|1158066	ASPNIDRAFT_174907	An02g01730; dicarboxylate transporter	K13577	193	4E-11	Aspergillus niger ATCC 1015 v4.0	Aspni7_GeneCatalog_proteins_20131226.aa
18		Aspni7|1168387	ASPNIDRAFT_42962	An18g05590; carrier protein YMC1; solute carrier family 25 (mitochondrial	K15109	180	4E-07	Aspergillus niger ATCC 1015 v4.0	Aspni7_GeneCatalog_proteins_20131226.aa
19		Aspni7|1137850	ASPNIDRAFT_50781	An03g06860; amino-acid transporter arg-13; mitochondrial ornithine carrier protein	K15114	180	5E-07	Aspergillus niger ATCC 1015 v4.0	Aspni7_GeneCatalog_proteins_20131226.aa
20		Aspni7|1011311	ASPNIDRAFT_205206	An04g08800; carrier protein LEU5; solute carrier family 25 (mitochondrial carrier protein), member 16	K15084	180	5E-07	Aspergillus niger ATCC 1015 v4.0	Aspni7_GeneCatalog_proteins_20131226.aa
21		Aspni7|1163839	ASPNIDRAFT_182498	An15g03200; thiamine pyrophosphate carrier 1	K15108	175	2E-06	Aspergillus niger ATCC 1015 v4.0	Aspni7_GeneCatalog_proteins_20131226.aa
22		Aspni7|1109905	ASPNIDRAFT_213553	mannan polymerase complexes MNN9 subunit [EC:2.4.1.232]	K05529	111	2E-07	Aspergillus niger ATCC 1015 v4.0	Aspni7_GeneCatalog_proteins_20131226.aa
23		Aspni7|1107040	ASPNIDRAFT_52427	An02g12070; phosphate carrier protein; solute carrier family 25 (mitochondrial phosphate transporter), member 3	K15012	109	3E-07	Aspergillus niger ATCC 1015 v4.1	Aspni7_GeneCatalog_proteins_20131227.aa

Supplementary Table 2. Accession numbers of the sequences for phylogenetic tree construction.

Species	Accession no.
Saccharomyces cerevisiae	YBR291C (*Sc*Ctp1p), YJR095W (*Sc*Sfc1p)<br>YFR045W (*Sc*SLC25A1)
Arabidopsis thaliana	9306999 (*At*SFC1)
Homo sapiens	6576 (*Hs*CTP)
Mus musculus	13358 (*Mm*Ctp)
Aspergillus nidulans	AN3461.2 (*An*SLC25A1.1), AN7287.2 (*An*SLC25A1.2)
Rattus norvegicus	29743 (*Rn*Ctp)
Aspergillus oryzae	AO090005000048 (*Ao*SLC25A1.1), AO090020000012 (*Ao*SLC25A1.2),<br>AO090023000454 (*Ao*SLC25A1.3), AO090102000125 (*Ao*SLC25A1.4)
Candida Albicans	CAALFM_C304270CA (CaCtp1), CAALFM_C504440CA (CaSfc1p)
Schizosaccharomyces pombe	SPAC19G12.05 (*Sp*SLC25A1)
Trichophyton benhamiae	ARB_03294 (*Tb*SLC25A1)
Trypanosoma cruzi	509561.30 (*Tc*Ctp)
Cavenderia fasciculata	DFA_09988 (*Cf*McfZ)
Clavispora lusitaniae	CLUG_01867 (*Cl*SLC25A1)
Sugiyamaella lignohabitans	AWJ20_4648 (*Sl*Sfc1p)
Auxenochlorella protothecoides	F751_5780 (*Ap*Sfc1)
Monoraphidium neglectum	MNEG_2923 (*Mn*Sfc1)
Oryza sativa japonica (*Japanese rice*)	4332510 (*Osa*Sfc1)
Lactuca sativa	111887930 (*Ls*Sfc1)
Ipomoea nil	109182393 (*In*Sfc1)

Vigna angularis	108336366 (*Va*Sfc1)
Crassostrea gigas	105344189 (*Cg*SLC25A1)
Populus trichocarpa	7460363 (*Pt*Sfc1)
Schistosoma mansoni	Smp_149320 (*Sm*Ctp1)
Tetranychus urticae	107362659 (*Tu*Ctp1)
Diuraphis noxia	107165760 (*Dn*Ctp1)
Nicotiana tomentosiformis	104114371 (*Nt*Sfc1)
Aspergillus niger	ASPNIDRAFT_136079 (CtpA), ASPNIDRAFT_42578 (CtpB), ASPNIDRAFT_194825 (CtpC), ASPNIDRAFT_52803 (CtpD), ASPNIDRAFT_41991 (CtpE), ASPNIDRAFT_174907 (CtpF)
Aspergillus luchuensis mut. kawachii	AKAW_03754 (*Ak*CtpA), AKAW_06280 (*Ak*YhmA)

Supplementary Table 3. Primers used in this study

Primers	Sequences (5´→3´)[a]
Primers used for amplification of *ctpA*-5'f sequence	
P3424	GGAATTCTCATATCTTGCACGTTTCCTTGTCT
P3425	CGGGATCCCTGGGAGACACTCAGCACTAGGAG
Primers used for amplification of *ctpA*-3'f sequence	
P3426	GCTCTAGAATACATTTGATGGGATACACTTTGG
P3427	AACTGCAGCATGGAACAGATGACCGAGGA
Primers used for amplification of *ctpB*-5'f sequence	
P3420	TGAATGCTCCGTAACACCCAGAATTCATTAGGAAGGAGTGGTGCTGGC
P3421	CATACATTATACGAAGTTATGGATCCCCGACGCCTTCTTGTTGTG
Primers used for amplification of *ctpB*-3'f sequence	
P3422	CTATACGAAGTTATTCTAGAACTAGTAAGGGTAGTTCAATGCGAC
P3423	CCAGTGCCAAGCTTGCATGCCTGCAGGAACACCCAATGCTACCTCCA
Primers used for amplification of *ctpC*-5'f sequence	
P3428	GTAACACCCAGAATTGAATTCTGTCTCGGTGGTAAAGGG
P3429	TTATGGATCCGAGCTGAGCTCTTCCATCATACCAGCACC
Primers used for amplification of *ctpC*-3'f sequence	

P3430	GCTATACGAAGTTATTCTAGAGCTGGACCTTTATTCACG
P3431	GCCAAGCTTGCATGCCTGCAGTCGCCAGCCATAGTGATT
Primers used for amplification of *ctpD*-5’f sequence	
P1779	GTAACACCCAGAATTGAATTCCTGGAGATGTAGGCTGTT
P1780	ACGAAGTTATGGATCGGATCCGAAGAAAGAGGGAAGAAGTGA
Primers used for amplification of *ctpD*-3’f sequence	
P1781	ACGAAGTTATTCTAGTCTAGATCCCGCATTCAGAACACC
P1782	GCAGGGGCCCACTAGACTAGTTTCCCAGTTTCCACTTTGCT
Primers used for amplification of *ctpE*-5’f sequence	
P1783	GCTCCGTAACACCCAGAATTCCAATGGCAATGACAGAAGC
P1784	GGATCCATAACTTCGGAGCTCAGATTCCCTCCGCCATTC
Primers used for amplification of *ctpE*-3’f sequence	
P1785	TTATTCTAGAACTAGACTAGTGGGTCCAAACATCCGTAA
P1786	TGCAGGCATGCAAGCCTGCAGAGGGCGAGACGACAAGAA
Primers used for amplification of *ctpF*-5’f sequence	
P3432	GTAACACCCAGAATTGAATTCGCCTTTGGTATCGGTTTG
P3433	TTATGGATCCGAGCTGAGCTCCAATCAGAGTCTCGGTTTCC
Primers used for amplification of *ctpF*-3’f sequence	
P3434	ACGAAGTTATTCTAGTCTAGAATTTGGCGAAGCATTACC

P3435	GCTTGCATGCCTGCACTGCAGGCCAGCCTCATAAACTCG
Primers used for confirmation of *ctpA* deletion	
P3416	CCCTATTGCCAACCCATCC
P3417	TCGGTGAATGGCTGGGTA
P3418	TACGTCTACCTACCCCTCGG
P3419	ATGGGACTTTGACGATGGAA
P641	CAATATCAGTTAACGTCGAC
P642	GGAACCAGTTAACGTCGAAT
Primers used for confirmation of *ctpB* deletion	
P1967	TCGGGACGATTACTGAACTTGG
P1968	GTTCCCTGCGAGCGATTAGA
P1969	TCGAGGCCATCCGTAACATAC
P1970	CCTAGTCATCTGGTCTCCCTCA
P641	CAATATCAGTTAACGTCGAC
P642	GGAACCAGTTAACGTCGAAT
Primers used for confirmation of *ctpC* deletion	
P2009	TGCTCGGAAGAATGACCT
P2010	ATCCGAACTTTGATGGTGTCTG
P2011	AAGCAGCCAACTTCACAG

P2012	AACGGTTGTTGAGAATGAGA
P641	CAATATCAGTTAACGTCGAC
P642	GGAACCAGTTAACGTCGAAT
Primers used for confirmation of *ctpD* deletion	
P2013	GCCTCGCTCGTATCCCTT
P2014	TTCTGGGTGTTGTTGTTCG
P2015	CCCACTCACAACAAACTAACCC
P2016	CTGGGATTGGTTGTCTCG
P641	CAATATCAGTTAACGTCGAC
P642	GGAACCAGTTAACGTCGAAT
Primers used for confirmation of *ctpE* deletion	
P2017	GGCTCTGGAGGAGATTGTT
P2018	GGAGTTGAACCGAGTCTGC
P2019	GCTGCCGACCTACTTCTT
P2020	TACCTGCTTGGAGGGAGT
P641	CAATATCAGTTAACGTCGAC
P642	GGAACCAGTTAACGTCGAAT
Primers used for confirmation of *ctpF* deletion	
P2021	GAGGCATACTTCCCGTTCA

P2022	ACCACCGAACCAGAAAGG
P2023	CAATCCGACGCTGCTCTT
P2024	AGTTCTACCACGCCTTCC
P641	CAATATCAGTTAACGTCGAC
P642	GGAACCAGTTAACGTCGAAT
Primers used for complementation *ctpA*	
P3407	AAGCATCATACGGGAGTCATCGTTGCTGCA
P3408	ATCCGAGCTCGAATTTAGGGTCAGGTATCG
Primers used for complementation *ctpB*	
P3405	AAGCATCATACGGGATGGCTTCATAGAAAC
P3406	ATCCGAGCTCGAATTTTTATTAACGCCTCG
Primers used for complementation *ctpC*	
P3409	AAGCATCATACGGGATGGCAGATGACGACA
P3410	ATCCGAGCTCGAATTTTCCTGCAGTCGAGG
Primers used for complementation *ctpD*	
P3411	AAGCATCATACGGGATATAAATGAATTGAT
P3412	ATCCGAGCTCGAATTTTACAACGGAAGTCT
Primers used for complementation *ctpE*	
P3413	AAGCATCATACGGGAACTTCTTGACTTGAC

P3414	ATCCGAGCTCGAATTCGCGTAGTAGCTTCC
Primers used for complementation *ctpF*	
P3415	AAGCATCATACGGGATTACAGCGACTGTGA
P3416	ATCCGAGCTCGAATTAATAAGCCAAAAATA
Primers used for RT-PCR of *ctpA*	
P3443	ATCCTGTCCCACCGAAAC
P3444	AAATCCTGCGCCGAAACC
Primers used for RT-PCR of *ctpB*	
P3441	AACAAGAAGGCGTCGGTGCT
P3442	ATGAAACAAATCGCACGG
Primers used for RT-PCR of *ctpC*	
P3445	TCACCTCCTACGAATCTTACA
P3446	GTCCGCCAAACTGTGATG
Primers used for RT-PCR of *ctpD*	
P3447	GCCGGTGAGGAATACTACAA
P3448	CGAACAGGTTGCGGTAGAAGG
Primers used for RT-PCR of *ctpE*	
P3449	GCACCCAGCACAACTACAA
P3450	CCATGCCCAGGTGCTTCA

Primers used for RT-PCR of *ctpF*	
P3451	TCGGTGGTTCAGCATCAT
P3452	GTGGTGGAGTAGGTCAGTTGG
Primers used for RT-PCR of *actA*	
P992	GCTCCTAGCGTCCTGGGTCT
P993	CCTTCTGCATACGGTCGGAGATA
Primers used for overexpressing of *ctpA*	
P3619	ATGGCAACCTCCGAG
P3620	TATCGCCGCA AAGAC
Primers used for overexpressing of *ctpB*	
P3617	ATGGCTACCCTTGCC
P3618	TCATTCAACCCCAGC
Primers used for overexpressing of *ctpD*	
P3621	ATGTCGAACAACAAC
P3622	GCCGGCCGAGGCGCG

[a] Underlined sequences denote the restriction enzyme recognition sites.

Supplementary Figure 1

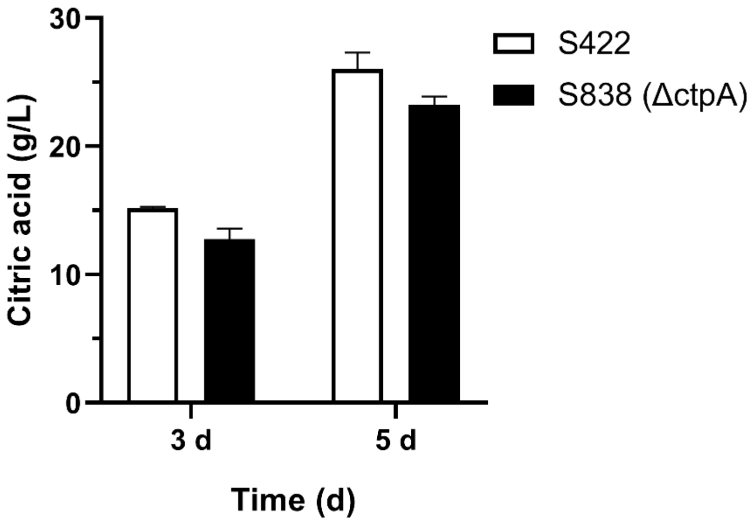

Supplementary Figure 2

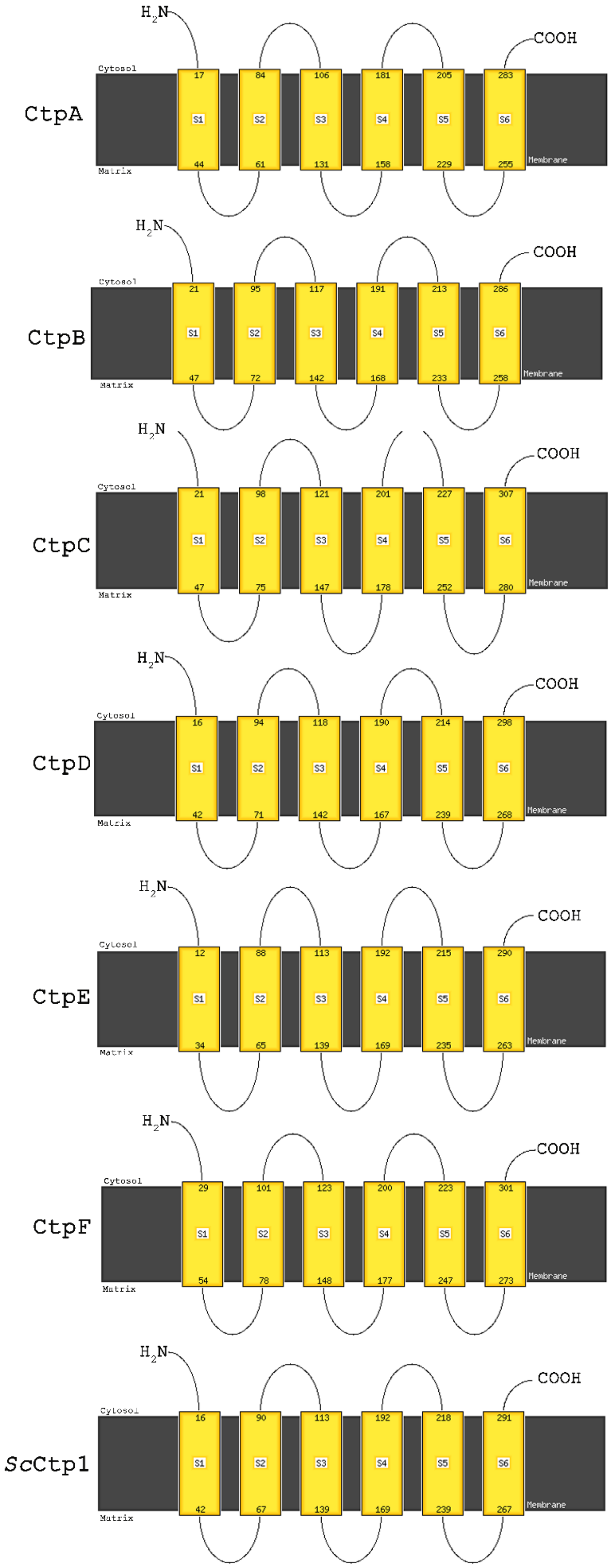

Supplementary Figure 3

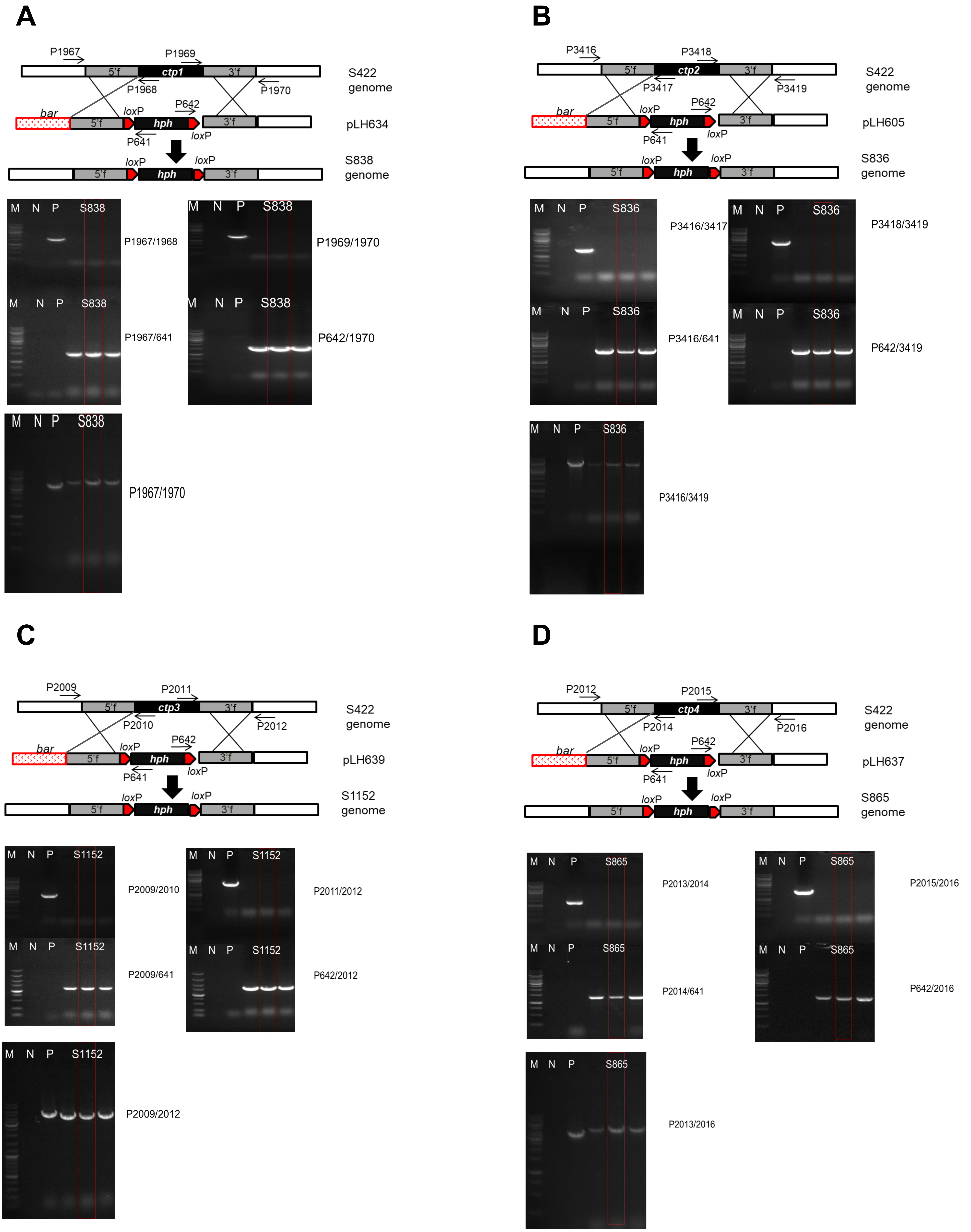

Supplementary Figure 3

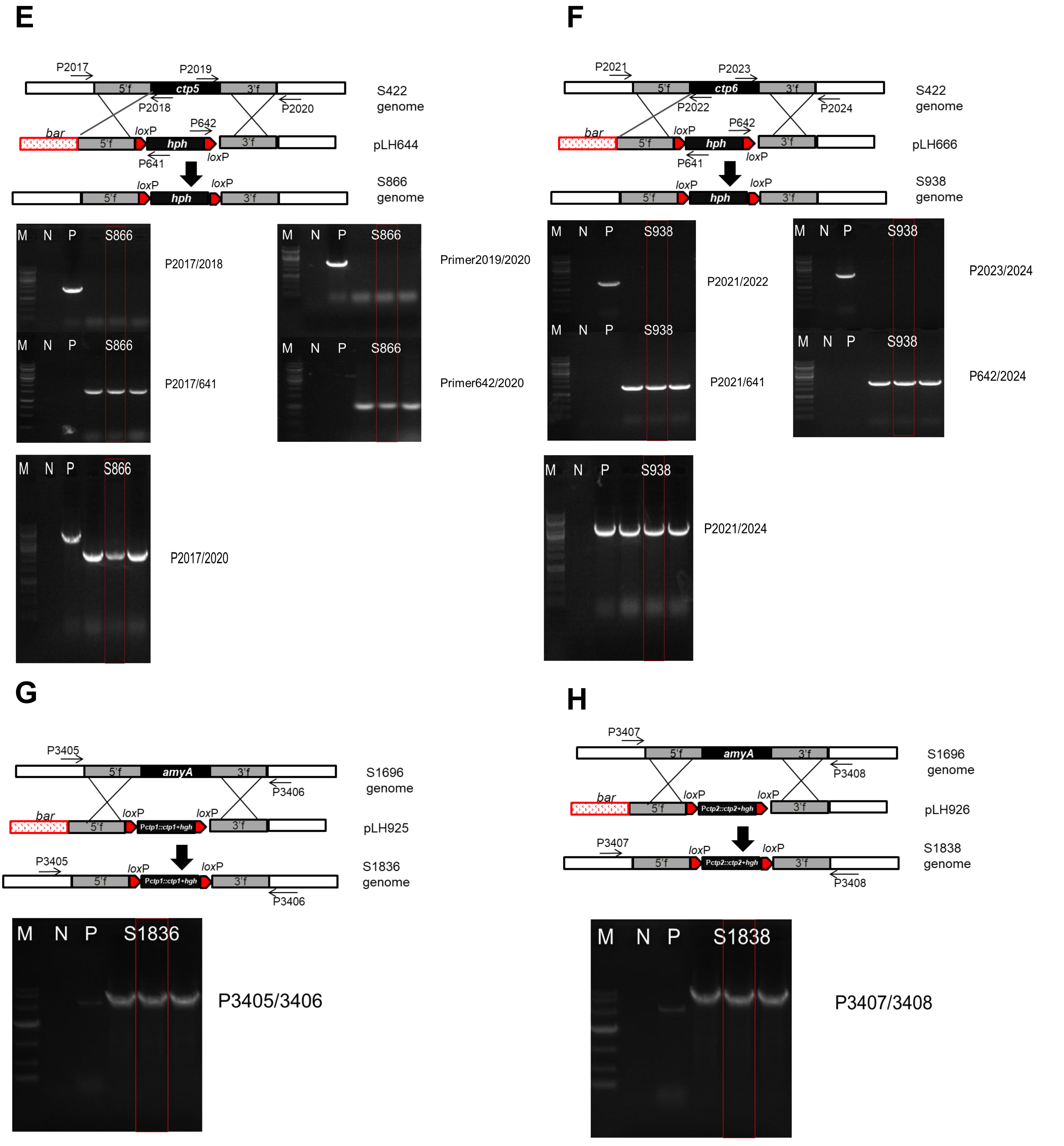

Supplementary Figure 3

I

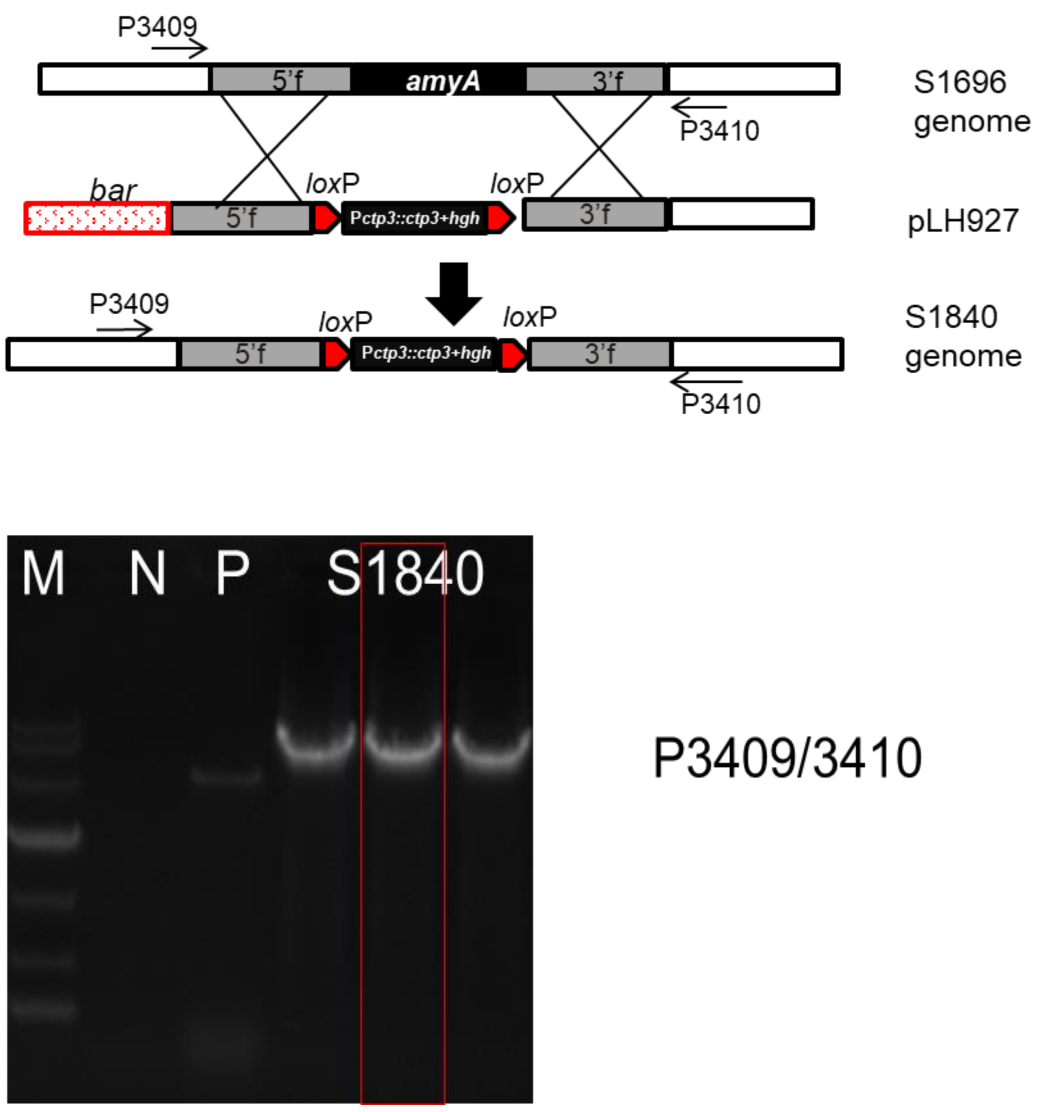

G

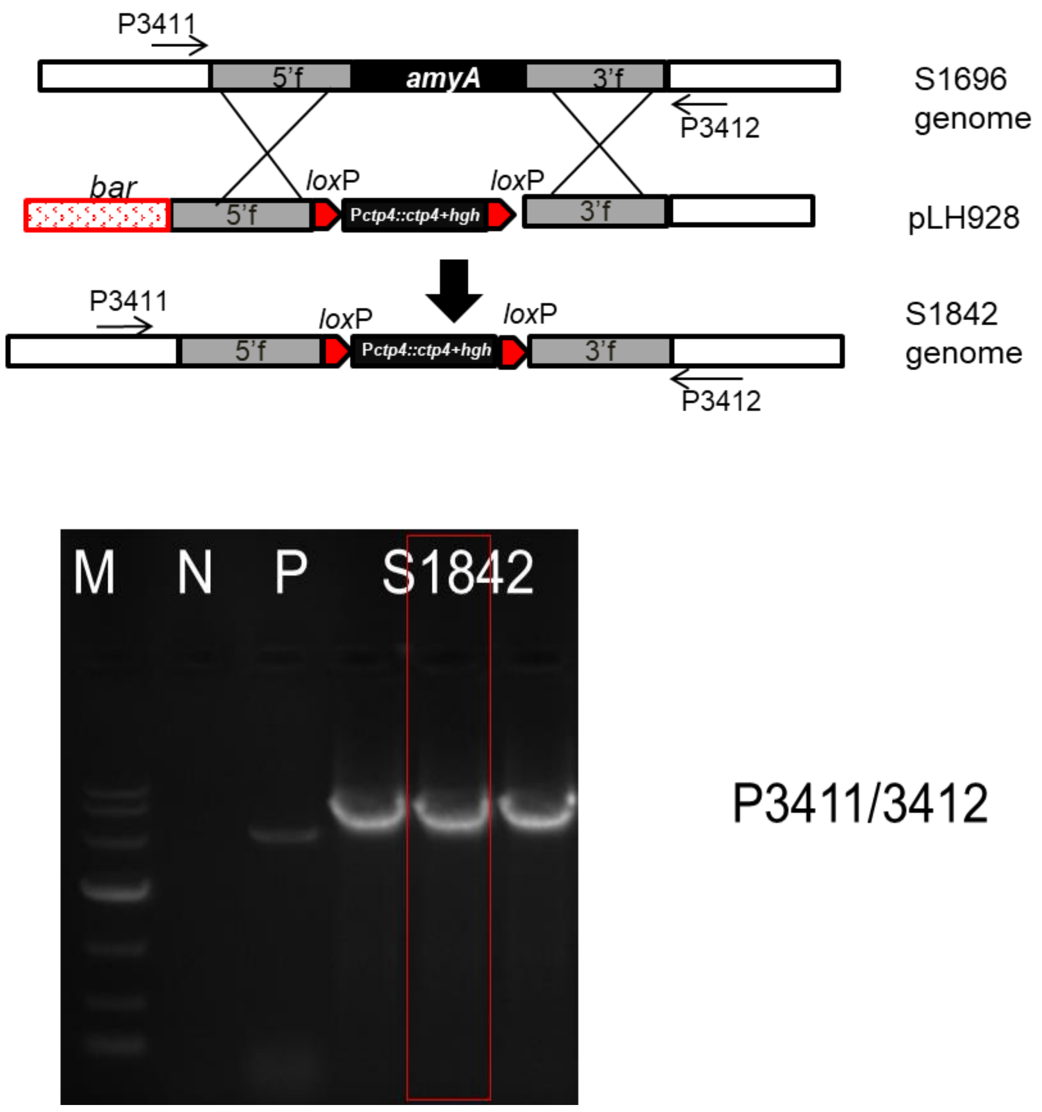

K

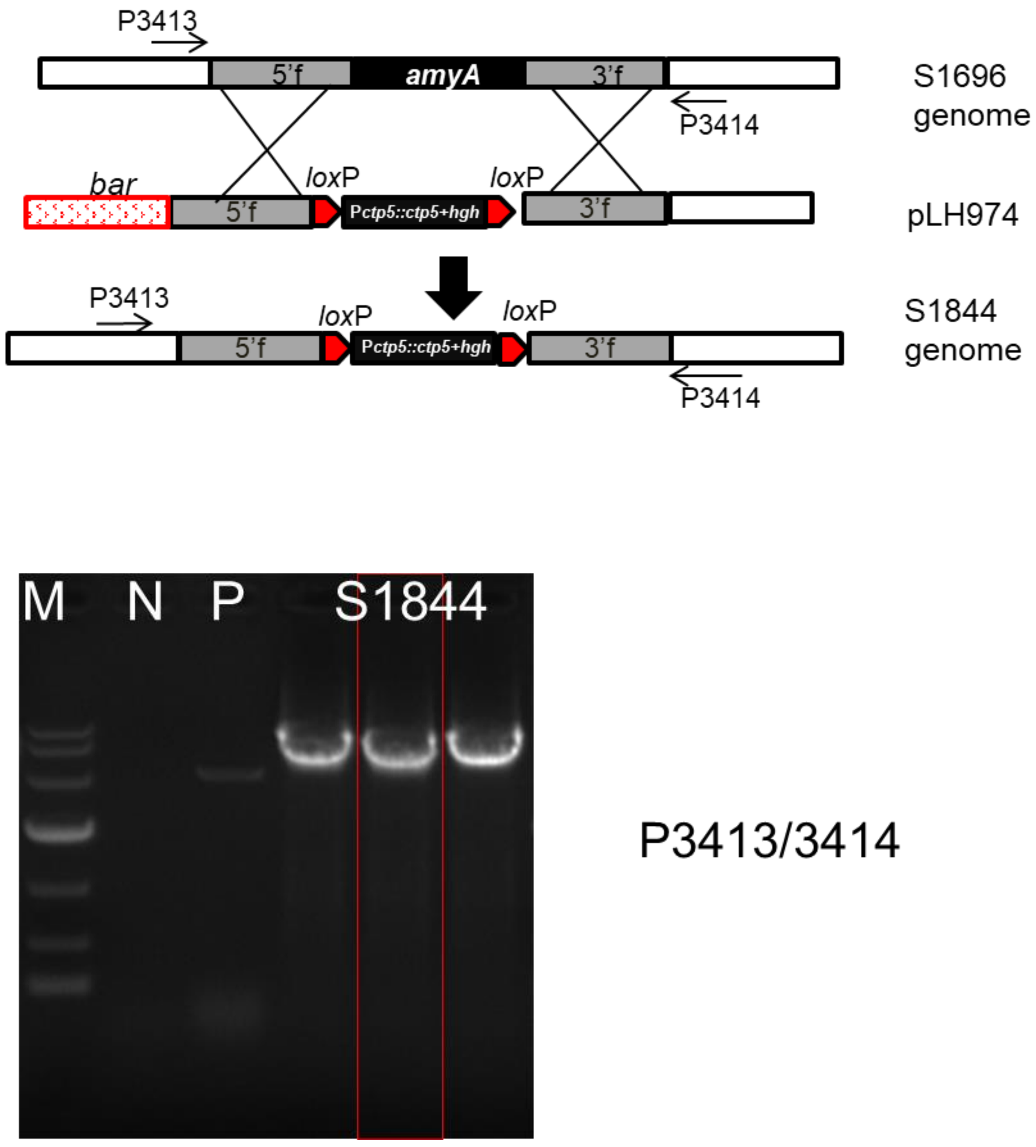

L

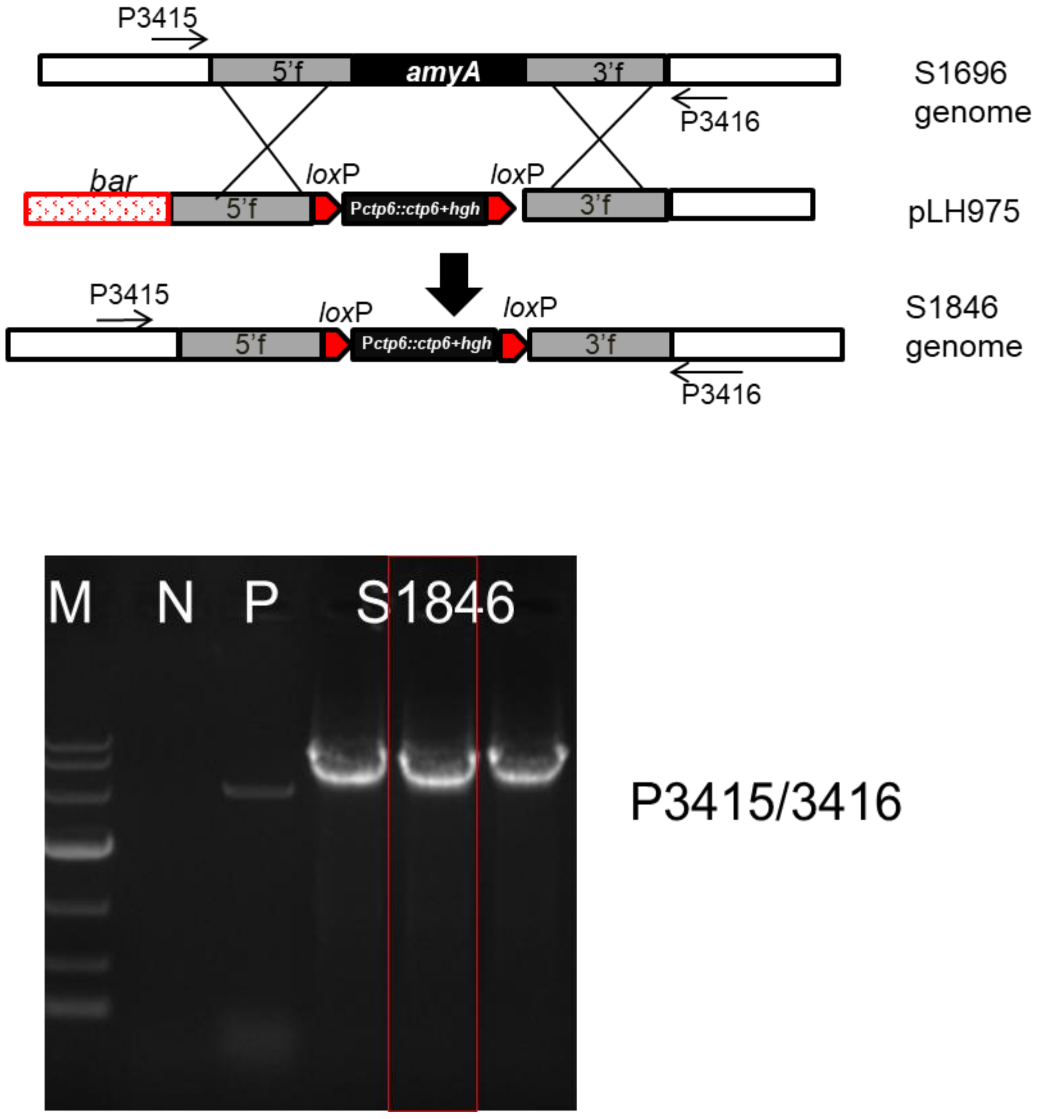

Supplementary Figure 3

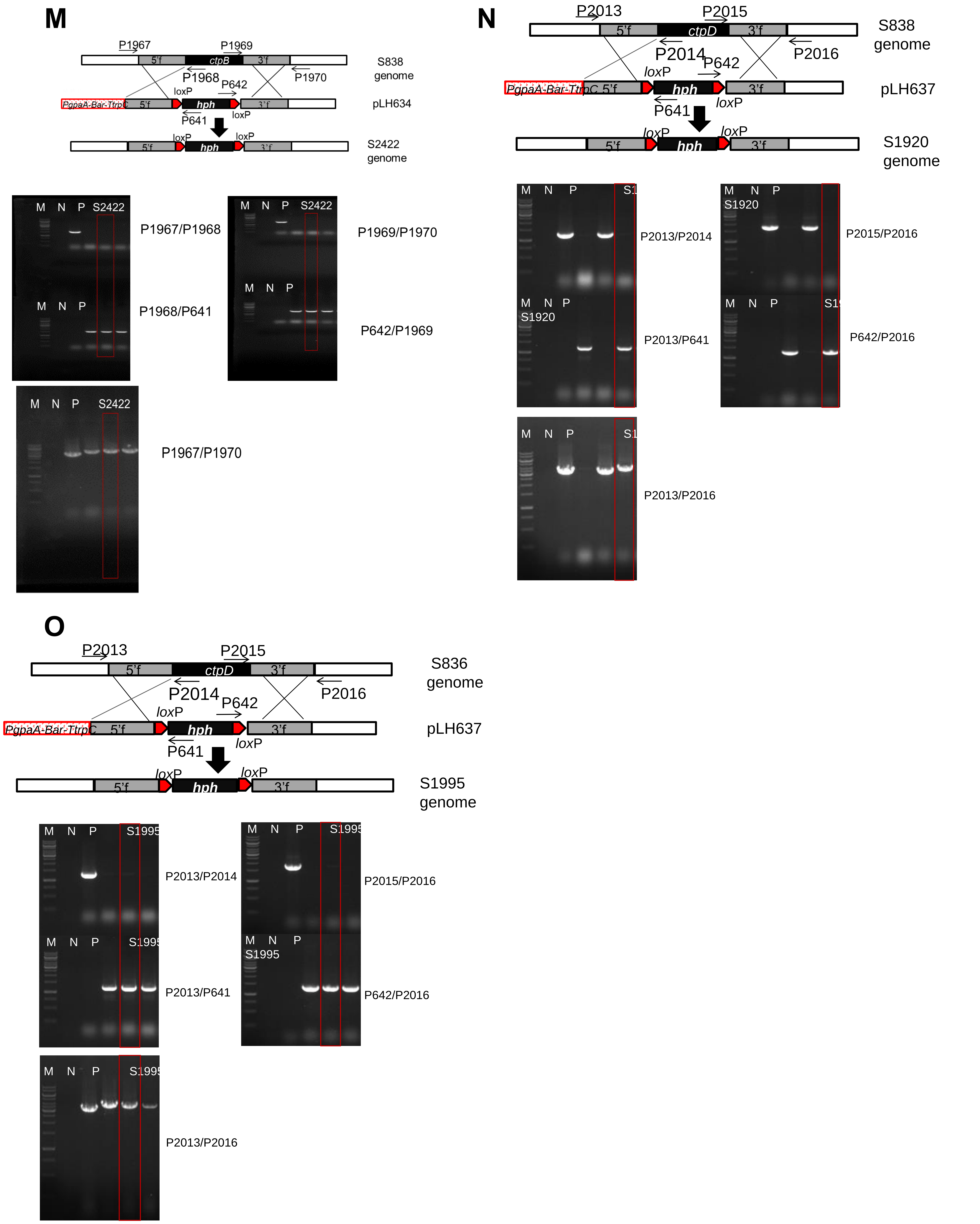

Supplementary Figure 4

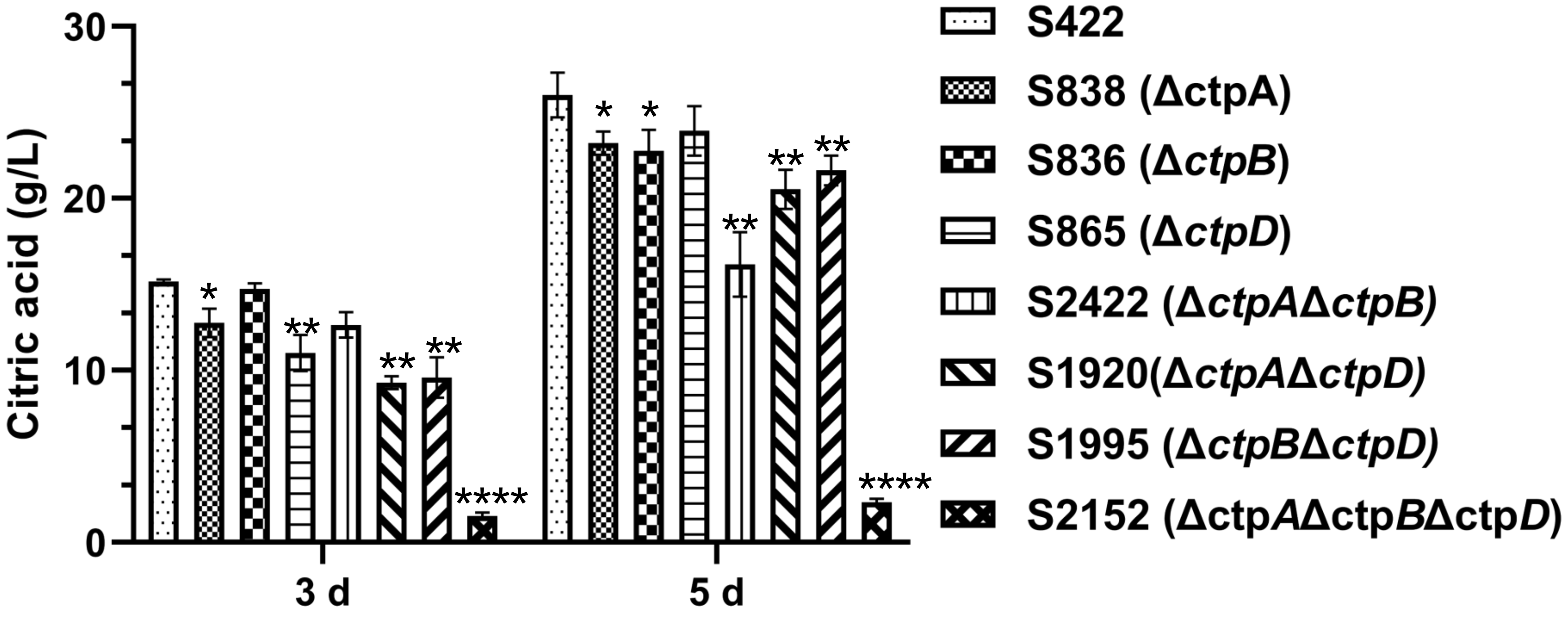

Figure Legends

Supplementary Figure 1 Citric acid production of *A. niger* S422 and S838 (Δ*ctpA*).

Supplementary Figure 2 Ctp1-Ctp6 and *Sc*Ctp1 transmembrane domain (TMD) were analyzed by Phyre2 web portal (http://www.sbg.bio.ic.ac.uk/phyre2/html/page.cgi?id=index) with the default paremeters.

Supplementary Figure 3 Construction of *A. niger* mutants used in this study.

Schematic of homologous recombination and the electrophoretic analyses of the verification PCR products for disruption of *ctp1*-*ctp6* (a-f) and individual reintroduction of *ctp1*-*ctp6* expression cassttee with their native promoter into S1696 (Δ*ctp1-6*) are shown in the panels (g-l).

Supplementary Figure 4 Citric acid productions of *A. niger* strains.

1×10^8 conidia of each *A. niger* strain as indicated were inoculated in 50 mL citric acid fermentation medium at 28°C for 3 d and 5 d, respectively. Citric acid titers were determined by HPLC.
